# Fuzzy-based multi-objective scheduling for human–robot manufacturing systems

**DOI:** 10.1038/s41598-026-40004-9

**Published:** 2026-02-27

**Authors:** Yijun Deng, Binrong Huang, Shouliang Lai

**Affiliations:** College of Packaging Design and Art, Hunan University of Technology, Zhuzhou, 412000 Hunan China

**Keywords:** Production planning, Human–robot interaction, Scheduling, Fuzzy programming, Multi-objective optimization, Engineering, Mathematics and computing

## Abstract

This study addresses the optimization of production planning and scheduling for human–robot interaction in a fuzzy environment, a critical challenge in modern manufacturing, especially under fluctuating market demand. The proposed model simultaneously determines production quantities, inventory/shortage levels, human–robot task allocation, and job sequencing. All decisions are optimized in a multi-period, multi-product setting. Three objective functions are considered: maximizing net present value, minimizing maximum completion time, and minimizing total early and tardy times. To handle uncertainties in demand and processing times, a pessimistic (credibility-constrained) fuzzy programming approach is employed. The model is solved using the epsilon-constraint method for small-scale problems and metaheuristic algorithms (NSGA-II, MOPSO, and MOWOA) for larger instances. Sensitivity analyses reveal that reducing completion times increases costs, lowering net present value, while higher uncertainty rates increase production times and shortages, reducing net present value. A 4% increase in bank interest rate reduces net present value by 15.68%, with no impact on completion or early/tardy times. The MOWOA algorithm demonstrates superior performance in generating efficient solutions for large-scale problems, offering practical insights for optimizing human–robot collaboration in manufacturing.

## Introduction

Today, the dynamics of businesses and competition among manufacturing companies have led to changes in product production strategies. Production strategy is actually the achievement of an appropriate production plan in order to effectively use available resources and schedule the use of resources^1^. The importance of scheduling has become increasingly important in recent years due to the increasing diversity in customer demand, the shortening of product life cycles, the rapid development of new processes and technologies, and the resulting rapid changes and fluctuations in competitive markets. These commercial and economic pressures of the market require a system that, despite minimizing inventory, is able to meet a high level of customer satisfaction with products and orders, so these systems require accurate and implementable schedules^2^.

One of the key resources in production planning is the combination of humans and robots (machines). The limited resources available in manufacturing companies compel planning managers to allocate humans and robots optimally to tasks. Each resource type exhibits distinct characteristics, such as processing speed, task suitability, and associated setup and processing costs, all of which must be taken into account during allocation decisions^3,4^. In addition to human–robot task assignment, the sequencing of tasks performed by the same resource represents another critical aspect that manufacturing companies must address^5^. The importance of production planning and human–robot scheduling in the production line is very important due to the different requests of customers. Producing products ahead of time leads to inventory accumulation and the company must pay for its maintenance costs^6^. Also, if the production of products is carried out later than the scheduled time, it will cause customer dissatisfaction and will also be subject to fines. Therefore, the production schedule should be such that in addition to delivering customer orders on time, resources are also used optimally and there is no interruption in the production line. Because the cost of restarting the production line and the cost of deploying robots is very high^7^.

What affects the production planning and human–robot scheduling issue is the type of customer orders (uncertain demand) and the uncertain processing time of each order. Today, the competitive environment has led to the production of products not being mass and integrated, and manufacturing companies are producing diverse products in low volume in accordance with customer requests. The uncertainty of the demand amount due to the limited capacity of resources in the production line affects the total costs. On the other hand, as mentioned, the diverse demand for products leads to the uncertainty of the exact amount of processing time, which can greatly increase production costs, human–robot resources are not used well, and customer orders cannot be delivered on time.

Production planning, scheduling, and sequencing form the core of manufacturing companies’ operations. New and changing market demands make production a challenge^8^, as companies must deliver high-quality products using the least possible resources and respond to uncertain market demands^9^. Therefore, the need for efficient production planning, scheduling, and sequencing has become a very important research area for companies and researchers in recent decades^10^. Therefore, this paper addresses the optimization of production planning and scheduling problems in human–robot interaction under fuzzy conditions.

A distinct research gap persists despite the expanding corpus of work on fuzzy uncertainty modeling, human–robot collaborative scheduling, and production planning. Few studies simultaneously combine (i) detailed shop-floor scheduling that simultaneously determines human–robot task allocation, resource-type-dependent processing times, and job sequencing in a combined shop-floor environment with (ii) mid-term multi-period, multi-product production planning (determining production quantities, inventory, and shortage/backorder levels). Moreover, when uncertainty is considered in human–robot collaboration systems, it is predominantly addressed through deterministic, robust, or stochastic approaches; epistemic uncertainty arising from lack of historical data—typical in customized or low-volume production—is rarely modelled using fuzzy sets. Based on the investigations carried out, no previous work has proposed an integrated model that simultaneously (a) incorporates fuzzy demand and fuzzy processing times that differ by resource type (human vs. robot), (b) employs a risk-averse pessimistic (credibility-constrained) fuzzy programming approach, and (c) optimizes three conflicting objectives: maximizing net present value (financial performance), minimizing makespan, and minimizing total earliness/tardiness. The present study fills this gap by designing and solving such a complete multi-objective fuzzy model, so providing both theoretical advances and practical decision assistance for modern human–robot production systems operating under high uncertainty.

The problem studied in this study is classified as a combined shop floor scheduling problem, which deals with issues such as assigning tasks to resources and prioritizing the processing of tasks. In such problems, there is a possibility of interruptions between tasks. In this paper, the resources used are divided into two categories: human and machine. In addition to considering the scheduling problem, the production planning problem is also considered simultaneously in this problem to bring the problem closer to the real world.

The goal of optimizing this problem is to reach important decisions for manufacturing companies in terms of determining the production quantity, determining the amount of accumulated inventory, optimally allocating human–robot to the production line, and sequencing activities by resources in the production line. In order to make optimal decisions, three objective functions are considered: "maximizing the net present value", "minimizing the maximum processing time", and "minimizing the sum of early and late times". Finally, the main goal of this paper is to present an optimal model for this production planning and scheduling problem of human–robot communication. Consequently, the present study seeks to test the following propositions:

- An integrated multi-objective model that simultaneously calculates production quantities, inventory/shortage levels, human–robot task allocation, and job sequencing delivers greater financial and operational performance compared with sequential or deterministic techniques.
- Pessimistic (credibility-constrained) fuzzy programming delivers stronger scheduling robustness and higher net present value under severe demand and processing-time uncertainty than classical deterministic or possibility-based fuzzy models.
- Among state-of-the-art multi-objective meta-heuristics (NSGA-II, MOPSO, MOWOA), the Multi-Objective Whale Optimization Algorithm (MOWOA) provides a wider and higher-quality Pareto front for large-scale human–robot production-scheduling issues.

While the literature has extensively examined production planning, maintenance-integrated scheduling, and human–robot task allocation independently, the majority of extant models remain deterministic or presume that uncertainty can be effectively captured by probability distributions. In real-world human–robot production systems especially those creating customized, low-volume, or highly diverse items historical data are typically poor or nonexistent, rendering stochastic techniques inadequate. Rather, uncertainty in consumer demand and processing times (which vary greatly depending on whether a task is carried out by a robot or a human operator) is mostly epistemic and is thus more accurately represented by fuzzy sets. Furthermore, current research on human–robot cooperation (HRC) scheduling usually treats production volumes, inventories, and shortages as given or set exogenously and concentrates exclusively on shop-floor-level decisions (task-to-resource assignment and sequencing). The considerable interdependencies between specific shop-floor scheduling and mid-term production planning decisions are ignored by this division, which results in less than ideal operational and financial performance. An integrated framework that simultaneously determines production quantities, inventory/shortage levels, human–robot task allocation, resource-dependent processing times, and job sequencing in a multi-period, multi-product environment under fuzzy demand and fuzzy processing times is therefore clearly needed.

To address these limitations, the present study presents a multi-objective fuzzy mathematical programming model that explicitly blends mid-term production planning with precise human–robot shop-floor scheduling. Trapezoidal fuzzy numbers are used to describe uncertain parameters, and a pessimistic (credibility-constrained) fuzzy programming approach is used to govern them, reflecting the risk-averse attitude common among industrial decision-makers. The model is the first to simultaneously optimize three conflicting objectives maximizing net present value, minimizing makespan, and minimizing total earliness/tardiness thereby providing a comprehensive decision-support tool for modern human–robot manufacturing systems operating under high epistemic uncertainty.

The analysis in the prior subsections highlights three significant limitations that restrict the effective implementation of human–robot collaborative systems in highly customized manufacturing settings with limited historical data. These limitations include: (1) the separation of mid-term production planning activities such as determining production volumes, managing inventory, and addressing shortages from specific shop-floor human–robot task assignment and sequencing; (2) the overreliance on deterministic or stochastic uncertainty models, which prove inadequate when probability distributions are difficult to reliably estimate; and (3) the lack of integrated models capable of simultaneously addressing financial objectives (net present value), operational goals (makespan), and delivery performance targets (earliness/tardiness) under conditions of epistemic uncertainty. To address these gaps, this study introduces a comprehensive multi-objective mixed-integer programming model. This model fully integrates multi-period, multi-product production planning with human–robot shop-floor scheduling, incorporates resource-specific fuzzy processing times and fuzzy demand through a risk-averse credibility-constrained fuzzy programming approach, and optimizes the three competing objectives critical to managerial decision-making. Details on the model formulation, defuzzification process, and solution methodology are provided in the subsequent section.

### Highlights (summarizing the major contributions)

- Presents the first integrated multi-period, multi-product production planning and detailed human–robot shop-floor scheduling model that simultaneously estimates production volumes, inventory/shortage levels, task-to-resource (human/robot) allocation, and job sequencing.
- Introduces a unique risk-averse pessimistic (credibility-constrained) trapezoidal fuzzy programming paradigm to handle epistemic uncertainty in demand and resource-type-dependent processing durations under limited or absent historical data.
- Simultaneously optimizes net present value (financial performance), minimizes makespan, and minimizes the total earliness/tardiness penalty three competing managerial goals.
- Shows that in 15 benchmark cases including an actual industrial packing situation, the Multi-Objective Whale Optimization Algorithm (MOWOA) performs noticeably better than NSGA-II and MOPSO in terms of Pareto front quality, variety, and proximity to the ideal point.
- Provides practical managerial insights on the trade-offs between robot deployment intensity, operational performance (makespan and earliness/tardiness), and financial consequences under demand and processing-time uncertainty.
- The present of the first complete simultaneous integration of detailed shop-floor human–robot collaborative scheduling (task-to-resource-type assignment R_pwht_ $\in${0,1} and job sequencing H_pp’w_ with mid-term multi-period, multi-product production planning (determining production quantities X_pt_, inventory levels Y_pt_, and backorder quantities S_pt_) in a single mixed-integer programming model solved jointly. While previous studies (e.g., Alimian et al., 2020; Liu et al., 2021; Ghaleb et al., 2021; Goli et al., 2023) have integrated production/inventory decisions with scheduling, they either (i) treat resource type (human vs. robot) as fixed or exogenous, (ii) only take into account machine-based systems without human operators, or (iii) model human–robot collaboration only at the shop floor level while fixing production volumes, inventory, and shortages exogenously^11–14^.

This research is organized into 6 sections. In the second section, a review of the research literature is reviewed and the research gap is expressed in the form of a comparative table. In the third section, a production planning and scheduling model for human–robot communication is presented, and the proposed pessimistic (credibility-constrained) fuzzy programming approach is used to control the uncertain parameters of demand and processing time. In the fourth section, problem solving methods including MOPSO (Multi Objective Particle Swarm Optimization), NSGA II (Non-dominated Sorting Genetic Algorithm II) and MOWOA (Multi Objective Whale Optimization Algorithm) algorithms are introduced and the initial solution of the problem is designed in accordance with the mathematical model. In the fifth section, the implementation of the mathematical model is discussed in the form of various numerical examples. The efficiency of meta-heuristic algorithms is examined in terms of various indicators in this section. In the sixth section, conclusions and suggestions for future research are presented.

## Research background

### Integrated production planning and scheduling

Production planning and scheduling are inherently hierarchical processes with a high degree of interdependence between decisions. Numerous studies highlight the advantages of integrating mid-term planning addressing aspects such as lot-sizing, inventory management, and shortage handling with shop-floor scheduling. For instance, Alimian et al. (2020)^15^ explored the integration of scheduling with preventive maintenance in dynamic cellular systems. Liu et al. (2021)^16^ combined process planning and scheduling through a blend of mixed-integer programming and hybrid evolutionary algorithms. Similarly, Ghaleb et al. (2021)^17^ focused on optimizing maintenance alongside flexible job-shop scheduling, while Gharoun et al. (2022)^18^ developed a framework for combining production scheduling with reliability-based maintenance planning. Goli et al. (2023)^19^ tackled non-permutation flow-shop scheduling with objectives centered on energy efficiency and cost reduction using MOWOA and related algorithms. Despite the success of these studies in bridging tactical and operational levels, none offer a comprehensive model that simultaneously accounts for production quantities, inventory or shortage management, human–robot task allocation, and detailed job sequencing.

Multi-objective scheduling techniques based on decomposition and metaheuristic optimization have shown notable gains in computing efficiency and solution quality for complex production systems in the context of sophisticated manufacturing environments^20^. In order to improve decision quality in dynamic production contexts with unpredictable work arrivals and real-time system fluctuations, hybrid knowledge-driven and data-driven scheduling techniques have also been proposed^21^.

### Human–robot collaborative scheduling

Recent research increasingly emphasizes task allocation and sequencing in human–robot collaboration (HRC) settings. Key contributions include Bogner et al. (2018)^11^, focusing on printed circuit board assembly, Casalino et al. (2019)^22^, which applied fuzzy programming to dual-arm robots, and innovative approaches by Hari et al. (2020)^23^, Yu et al. (2021)^24^, and Maderna et al. (2022)^25^ addressing dynamic task sequencing and reinforcement learning. Vital-Soto et al. (2023)^12^ explored flexible job-shop scheduling with dual-resource constraints utilizing NSGA-II, while Vahedi-Nouri et al. (2024)^13^ and Khadivi et al. (2024)^14^ investigated reconfigurable systems and parallel-machine environments incorporating personnel constraints. Although these studies excel in optimizing shop-floor human–robot allocation and sequencing, they largely treat production volumes, inventory, and shortages as external or fixed variables, thus creating a disconnect between financial and operational performance integration.

Furthermore, in order to enhance operational efficiency and facilitate intricate automated production activities, intelligent process planning and path optimization approaches have been used more and more in contemporary manufacturing^26^.

### Uncertainty handling in production and HRC systems

The majority of early models for HRC scheduling are deterministic. Stochastic programming or resilient optimization is more common when uncertainty is taken into account^27^. Fuzzy techniques remain rare and are often limited to assembly-line balance or single-period problems. None of the current fuzzy HRC studies use a risk-averse credibility-constrained (pessimistic) framework appropriate for epistemic uncertainty arising from limited historical data to simultaneously model (i) multi-period production planning, (ii) resource-type-dependent fuzzy processing times, and (iii) fuzzy demand. The significance of sophisticated uncertainty representations that go beyond traditional probabilistic assumptions has been highlighted by recent multidisciplinary research that have also investigated the function of intricate statistical structures inspired by cognitive processes for simulating uncertainty and decision behavior in complex systems^28^.

### Multi-objective metaheuristic techniques for scheduling problems

Swarm intelligence and multi-objective evolutionary algorithms are frequently used to solve challenging scheduling issues. Bazargan-Lari et al. (2022)^29^ and Vital-Soto et al. (2023)^12^ utilized NSGA-II; Goli et al. (2023)^19^ used MOPSO and MOWOA for flow-shop issues; Baroud et al. (2023)^30^ presented hybrid ant-colony/PSO methods; and Yu et al. (2024)^31^ employed knowledge-based greedy algorithms. For large-scale scheduling instances, comparative investigations regularly demonstrate that whale-optimization-based algorithms frequently dominate in terms of convergence speed and Pareto-front diversity.

### Explicit definition and critical synthesis of the research gap

Despite the substantial growth of the literature on shop-floor scheduling, production planning, and human–robot collaboration (HRC), a critical synthesis identifies three fundamental weaknesses that the current work directly tackles.

First, the vast majority of HRC scheduling models take production volumes, inventory levels, and shortages as exogenous parameters or solve them sequentially rather than simultaneously with specific shop-floor decisions (task-to-resource assignment and sequencing). Works such as Bogner et al. (2018)^11^, Hari et al. (2020)^23^, Maderna et al. (2022)^25^, Vital-Soto et al. (2023)^12^, Vahedi-Nouri et al. (2024)^13^, and Khadivi et al. (2024)^14^ focus exclusively on shop-floor-level human–robot task allocation and sequencing, while mid-term production planning is either ignored or handled separately. This decoupling neglects the strong relationship between financial performance (influenced by inventory holding, shortage penalties, and robot deployment costs) and operational performance (makespan and earliness/tardiness), often resulting to poor solutions in practice.

Second, when uncertainty is taken into account in HRC systems, it is typically modeled using robust optimization or stochastic programming, which call for precise uncertainty sets or trustworthy probability distributions^27^. In low-volume, highly customized, or recently founded human–robot cells, historical data are scant, making epistemic uncertainty more appropriately represented by fuzzy sets. While several recent studies have started to apply fuzzy logic to human–robot scheduling including “The fuzzy human–robot collaboration assembly line balancing problem”^32^, and additional work by Hashemi-Petroodi et al. (2024)^33^, Raatz et al. (2024)^34^, and Jia et al. (2025)^35^ these studies are primarily focused on assembly line balancing or single-period tactical scheduling and do not incorporate multi-period production planning alongside financial goals.

Third, no prior study has used a risk-averse (pessimistic/credibility-based) fuzzy programming framework to simultaneously optimize three competing but managerially crucial goals: minimizing makespan, maximizing net present value (a direct financial measure that accounts for time-value of money, robot deployment costs, inventory holding, and shortage penalties), and minimizing total earliness/tardiness under fuzzy demand and resource-type-dependent fuzzy processing times.

Table 1 reviews some of the most important articles in the field of production planning and job scheduling.

**Table 1:** Review of previous research.

Author	Objective function(s)	Model feature	Model decisions	Human–robot communication	Model environment	Problem solving method
^36^	Delay	Spe-Spr	A-S	*	D	H
^11^	Profit	Spe-Spr	A	*	D	M-E
^37^	Cmax	Spe-Spr	S		F	M
^22^	Cost	Spe-Spr	A	*	F	H
^38^	Energy-Noise-Efficiency	Spe-Mpr	S		D	M
^39^	Cmax	Spe-Spr	S		D	M
^23^	Cmax	Spe-Spr	A-S	*	D	H
^40^	Cmax	Spe-Spr	A-S	*	D	H
^41^	Cost	Mpe-Mpr	A-S-P	*	D	E-S
^30^	Cmax	Spe-Spr	A-S		D	M
^42^	Profit-Energy	Spe-Mpr	A-P	*	D	M
^43^	Cost-Cmax	Spe-Mpr	A-S	*	S	E
^13^	Cmax	Mpe-Mpr	A	*	D	E
^44^	Cost	Spe-Spr	A-S-P	*	D	E
^27^	Cmax	Spe-Spr	A-S	*	S	E
^45^	Energy	Spe-Spr	A-S	*	D	E
Current research	NPV- Cmax- ELtime	Mpe-Mpr	A-S-P-Q	*	F	E-M

NPV (Maximizing net present value); Cmax (Minimizing the maximum completion time of tasks); Profit (Profit maximization); Cost (Cost minimization); Delay (Minimizing the delay between two consecutive tasks); Energy (Minimizing energy consumption); Noise (Minimizing noise pollution); Efficiency (Increasing production efficiency); EL time (Minimizing the sum of early and late times); D (Definite); F (Fuzzy); S (Contingency planning); M (Meta-innovative); E (Exact solution); H (Innovative); S (Simulation); A (Task allocation); S (Sequence of tasks); P (Determining production quantity); Q (Determine the inventory amount); Spe (Single- period); Mpe (Multi- period); Spr (Single product); Mpr (Multi-product).

A review of the literature on production planning and scheduling of human–robot interaction in production shows that most of the research has been conducted without considering production planning and inventory control issues. Also, most of the research has been conducted in a deterministic environment and fewer researchers have turned to providing non-deterministic models and meta-heuristic solution methods. Considering conflicting objective functions such as maximizing net present value, minimizing maximum completion time, and minimizing the sum of early and late times has been studied in this paper. Therefore, the innovations of the present paper can be summarized as follows:

- Presenting an integrated model of production planning and human–robot scheduling in the combined shop floor flow scheduling problem.
- Simultaneous consideration of decisions on assigning tasks to humans and robots, sequencing tasks, determining production quantities, and determining inventory quantities in the mathematical model.
- Controlling uncertain demand and processing time parameters with pessimistic fuzzy programming method.
- Using NSGA-II, MOPSO, and MOWOA algorithms with initial solution design to solve the problem.

## Research methodology

Considering the research gap in this section, a model for the production planning and scheduling problem of human–robot interaction under uncertainty and control of uncertain parameters of demand and processing time has been presented using the proposed pessimistic (credibility-constrained) fuzzy programming method. The presented model includes a set of available resources (human–robot) that seek to produce sets of products in different time periods at each workstation. The main goal is to allocate products for production to resources (human–robot) and the sequence of operations by these resources. Also, the optimal amount of product production in each time period is determined based on the demand for uncertain products. So that there is the possibility of storing the produced products and also a shortage in meeting the demand simultaneously in each time period. Which product should be produced at which workstation and by what type of resource is an important issue that we will answer in the mathematical model? In this research, the processing time and the amount of product demand are considered uncertain.

The assumptions of the presented mathematical model are as follows:

- The problem is multi-period and multi-product.
- Every product must be processed sequentially through all workstations (hybrid flow-shop structure).
- Workstations are universal: Any product can be processed at any workstation by either a human operator (h = 0) or a robot (h = 1). This premise aligns with modern collaborative manufacturing setups, where multi-functional cobot-type robots are installed at all workstations and human operators are either cross-trained or work collaboratively with robots without any task-specific limitations. For the industrial scenario that inspired this study a packaging manufacturer in Hunan Province all 18 workstations are equipped with collaborative robots capable of handling every required operation. Additionally, human operators are trained to manage any product across all stations. There are no technological or skill-based constraints that would restrict any specific combination of human, robot, product, or workstation. Consequently, the key differences between resource types are limited to processing speed, cost, and availability, all of which are explicitly incorporated into the model using resource-type-dependent fuzzy processing times, denoted as ${\widetilde{\delta }}_{pwh}$, along with their respective costs.
- Shortages are fully back-ordered (no lost sales).
- Processing times and demand are uncertain and represented by trapezoidal fuzzy numbers.
- Setup times are sequence-independent or negligible and are already included in the given processing times.
- Preemption is not allowed.
- The number of available robots per period is limited and incurs fixed and variable deployment costs.
- Inventory holding and shortage costs are linear.
- The planning horizon is finite and known.
- Shortages are explicitly allowed and managed as backorders, ensuring no sales are lost. Any unmet demand during period t, due to limitations in current production or available inventory, is automatically carried forward to be fulfilled in subsequent periods. The shortage amount for product p at the end of period t, represented as S_pt_ ≥ 0, is defined as a continuous (non-binary) decision variable. This variable is determined within the inventory-balance constraint outlined in Eq. 32 of the deterministic-equivalent model:
  I_pt_ + Y_p,t-1_ – Y_pt_ + S_pt_ – S_p,t-1_ = ${\widetilde{\tau }}_{pt}^{\alpha }$ (crisp equivalent after applying the credibility constraint with level α), with initial Y_p0_ = S_p0_ = 0.
- A linear per-unit, per-period backorder penalty cost, denoted as μ_pt_, is applied for each unit of shortage during each period. This penalty impacts the net present value in the first objective function by contributing to the reduction through the term
  $$- \sum_{t}\sum_{p}\frac{{\mu }_{pt}{S}_{pt}}{{\left(1+i\right)}^{t}}$$

The model does not implement a binary mechanism to permit or prevent shortages. Instead, backorders arise naturally whenever production and inventory levels fail to meet the credibility-constrained demand. Their financial impact is clearly and transparently accounted for using the linear penalty coefficient μpt.

A pessimistic (credibility-constrained) fuzzy programming method is utilized to convert the fuzzy parameters, specifically demand ${\widetilde{\tau }}_{pt}$ and processing times ${\widetilde{\delta }}_{pwh}$, into their crisp equivalents. This approach is intentionally risk-averse, and its rationale can be explained as follows:

- The epistemic nature of uncertainty becomes evident in low-volume, highly customized, or newly established human–robot cells, such as the real packaging factory case analyzed. In these scenarios, reliable historical data are either limited or entirely unavailable. As a result, it is difficult to estimate probability distributions with confidence, which are essential for stochastic programming. This makes it more suitable to model epistemic uncertainty using fuzzy sets instead of aleatory (random) distributions.
- Risk-averse management tendencies: Industrial decision-makers often focus on maintaining schedule stability and controlling costs rather than pursuing uncertain benefits. Approaches driven by optimism, relying on possibilities, generally result in overly ambitious plans that often breach constraints under unfavorable conditions. Methods like centroid/defuzzification or expected-value approaches, which average outcomes across the membership function, fail to provide a formal safeguard against adverse scenarios.
- The credibility measure, defined as Cr {⋅} = (Pos {⋅} + Nec {⋅})/2^46^, serves as a balanced pessimistic criterion by blending elements of pure optimism (Possibility, Pos) with extreme conservatism (Necessity, Nec). By requiring all fuzzy constraints to meet a minimum credibility threshold of α ≥ 0.5 (commonly set at 0.8 in our experiments), the resulting crisp equivalents ensure that each constraint is satisfied with at least α credibility. This means the decision-maker commits to a plan only if the belief in its feasibility meets or exceeds this level. Such an approach offers stronger safeguards against unfavorable outcomes compared to possibility-based models while avoiding the excessive rigidity and costs associated with necessity-based models.
- Empirical evidence of superiority within the problem context is demonstrated through sensitivity analyses (refer to Table 11 and the associated discussion). These analyses verify that increased α values consistently decrease net present value variability while mitigating significant shortages and delays under worst-case scenarios. This outcome supports the adoption of a risk-averse approach for practical industrial application.

The credibility-constrained pessimistic approach effectively aligns with the nature of epistemic uncertainty while addressing the practical need for robust and actionable production plans in human–robot manufacturing systems that operate with limited historical data.

To model the production planning and scheduling problem of human–robot communication, the following symbols have been defined:

Sets and indices

- p, *p*′ $$\in$$ P Products *p*^*a*^, *p*^*b*^, …
- t, *t*′ $$\in$$ T Time periods *t*^1^,*t*^2^,…
- w $$\in$$ W Workstations *w*^1^, *w*^2^, …
- r Robots *r*^1^, *r*^2^, …
- h Allocated resource type ℎ^0^, ℎ^1^.
- h $\in$ H = {0,1} Resource type (0 = human, 1 = robot).
- r $\in$ R Individual robots (used only when counting total robots per period).

Subset

- *w*_*p*_ Workstation w for product p.
- *w*_*p*_^*init*^, *w*_*p*_^*last*^ First/last workstation w for producing product p.

Parameters

- θ_*t*_ Time available for each robot in period t.
- φ_*wt*_ Time available for each workstation w in time period t.
- δ̃_*pw*ℎ_ Processing time of product p at workstation w based on resource type h.
- τ̃_*pt*_ Demand for product p in period t.
- *k*_*pt*_ Selling price of product p in time period t.
- ϑ_*pt*_ Cost of producing product p in period t.
- α_*pt*_ Inventory cost of product p in period t and α is also used for a fuzzy confidence level.
- μ_*pt*_ Cost of shortage of product p in period t.
- π_*t*_ Cost of allocating the robot in period t.
- σ_*pw*_ Cost of allocating a robot to perform the production of product p at workstation w.
- ϖ_*pw*ℎ_ Resource type h for executing production of product p on workstation w.
- *B*_*t*_^*min*^, *B*_*t*_^*max*^ Minimum/maximum number of robots in period t.
- *K* Time horizon planning.
- *D*_*pw*_ Delivery time of product p at workstation w.
- *i* Bank interest rate.
- β_*pt*′*t*_ The effective ratio of the number of products p after supply period *t*′ ≤ *t* (Loading rate).
- ζ_*wt*_ Average occupancy/utilization ratio of workstation w in time period t.

Decision variable

- *I*_*pt*_ Quantity of product p supplied in period t.
- *X*_*pt*_ The effective quantity of product p to produce in period t.
- *Y*_*pt*_ Inventory of product p at the end of period t.
- *B*_*t*_ The total number of multi-purpose robots in period t.
- *R*_*pw*ℎ*t*_ 1; If resource type ℎ is allocated to produce product *p* at workstation *w* in period *t*.
  0; Otherwise.
- *C*_*pw*_ Time to complete product *p* at workstation w.
- *U*_*max*_ Time to complete product production at the last station.
- Η_*pp*′*w*_ 1; If product *p* is processed before product *p*′ at workstation *w*.
- 0; Otherwise.
- *E*_*pw*_ Early delivery time of product *p* at workstation *w.*
- *T*_*pw*_ Late delivery time of product *p* at workstation *w.*
- Η_*pp*′*w*_ Sequencing variable.
- R_pwht_ Resource-assignment variable.
- w_p_​, $${w}_{p}^{init}$$​, $${w}_{p}^{last}$$​ Workstation subsets.
- ${\widetilde{\delta }}_{pwh}$ Fuzzy processing time vectors.
- ${\widetilde{\tau }}_{pwh}$ Fuzzy demand vectors.

According to the defined symbols, the random programming model for the production and scheduling of human–robot communication under uncertainty is as follows:

1 $$\text{Max NPV }=\sum_{t}\frac{\sum_{p}\begin{array}{c}\left(kptXpt-\left(\vartheta ptXpt+\alpha ptYpt+\mu ptSpt\right)\right)- \\ {B}_{t {\pi }_{t}}-\sum_{p}\sum_{w}\sigma pwRpw{h}^{1}t\end{array}}{{(1+i)}^{t}}$$

2 $$Min\;C\max \, = U_{\max }$$

3 $$\text{Min ELtime }=\sum_{p}\sum_{w}({E}_{\mathrm{pw}}+ {T}_{\mathrm{pw}})$$

4 $$\begin{aligned}&\mathrm{s.t:}\\ & \sum\limits_{p} {\sum\limits_{h} {\sum\limits_{{t^{\prime } \le t}} {\beta _{{pt^{\prime } t}} } } } I_{{pt^{\prime } }} R_{{pwht}} \tilde{\delta}_{{pwh \le \zeta _{{wt}} \varphi _{{wt}} }}\quad \forall w,t \end{aligned}$$

a

5 $$X_{pt} = \sum\limits_{t^{\prime} \le t} {} \beta_{pt^{\prime}t} I_{pt^{\prime}} , \quad \forall p,t$$

$$Y_{{pt}} - S_{{pt}} = X_{{pt}} - \tau _{{pt}}+ Y_{{pt - 1}} - S_{pt - 1} ,\quad \forall p,t$$

Shortage costs are now explicitly incorporated into the NPV objective:

6 $$\sum_{p,t}{\mu }_{pt}{S}_{pt}$$

7 $$\sum\limits_{p} {\sum\limits_{w} {\sum\limits_{{t^{\prime } \le t}} {\beta _{{pt^{\prime } t}} } } } I_{{pt^{\prime } }} R_{{pwht}} \tilde{\delta}_{{pwh \le \theta _{t} {\mathrm{B}}_{t} }} \quad \forall t,h = 1$$

8 $$\sum_{h}{R}_{pwht=1} \quad\forall p,t,w\in wp$$

9 $$\mathop \sum \limits_{h} I_{pt} \le \mathop \sum \limits_{h} \tilde{\tau }_{pt} ,\;\;\;\forall p$$

10 $$B_{t}^{min} \le B_{t} \le B_{t}^{max} ,\;\;\;\forall {\mathrm{t}}$$

11 $$C_{pw} \, \ge \mathop \sum \limits_{h} \varpi_{pwh} \tilde{\delta}_{pwh} , \;\;\forall p,w \in w_{p}^{init}$$

12 $$C_{{pw}} \ge C_{pw - 1} + \sum\limits_{h} {\varpi _{{pwh}} \tilde{\delta }_{{pwh}} ~,~~\forall p,w \notin ~w_{p}^{{init}} }$$

13 $$C_{{pw}} \ge C_{p'w - 1} + \sum\limits_{h} {\varpi _{{pwh}} \tilde{\delta }_{{pwh}} ~ - {\mathrm{K}}(1 - {\mathrm{H}}_{{{\mathrm{p}}p'{\mathrm{w}}}} ),~~\forall w,p'> p}$$

14 $$C_{{p' w}} \ge C_{{pw}} + \sum\limits_{h} {\varpi _{{p^{\prime } wh}} } \delta _{{p^{\prime } wh}} - {\text{K }}({\mathrm{H}}_{{\mathrm{p}p' \mathrm{w}}}),\;\;\;\forall w,p'> p$$

15 $$U_{max} \ge C_{pw} ,\;\;\;\forall p,w \in w_{p}^{last}$$

16 $$E_{pw} = max[D_{pw} - C_{pw} ,0],\;\;\;\forall p,w \in w_{p}^{last}$$

17 $${\mathrm{T}}pw = max[{\mathrm{C}}_{pw} - {\mathrm{D}}_{pw} ,0],\;\;\;\forall p,w \in w_{p}^{last}$$

18 $$I_{pt} ,X_{pt} ,Y_{pt} ,S_{pt} ,B_{t} ,C_{mw} ,U_{max} ,E_{pw} ,T_{pw} \ge \, 0$$

19 $$R_{pwht} ,{\rm H}_{\mathrm{p}p^\prime w} \in \, \left\{ {0,{1}} \right\}$$

Equation (1) maximizes the net present value based on the bank interest rate. In this equation, the revenue from product sales is deducted from the current costs in each period. Equation (2) minimizes the maximum processing time of all jobs at the last workstation. Equation (3) minimizes the sum of the early and late times resulting from product delivery. Equation (4) ensures that the number of products produced by each type of resource must be less than the average employment ratio of that station. Equation (5) calculates the effective number of products produced based on the loading rate. Equation (6) is an equilibrium relationship of the amount of production, shortage and storage of products produced in each time period. Equation (7) limits the number of products produced by each robot in each workstation. Equation (8) shows that only one type of resource can be engaged in production in each workstation (human or machine). Equation (9) The amount of product supplied in all periods is less than the amount of demand in all periods. Equation (10) limits the minimum and maximum number of robots used in each period. Equations (11) to (14) calculate the time to complete the production of products at each workstation by a human or a robot. Equation (15) shows the time to complete the work at the last station. Equations (16) and (17) determine the early or late delivery of products relative to the definite delivery time. Equations (18) and (19) show the types of decision variables.

### Rationale for pessimistic fuzzy programming under epistemic uncertainty

When historical data are insufficient or non-existent a common circumstance in human–robot cells generating customized or low-volume products probability distributions cannot be properly predicted, rendering stochastic programming inappropriate. There are three primary credibility criteria for fuzzy approaches: possibility (optimistic), need (very conservative), and credibility (pessimistic). Possibility-based (optimistic) models^22^ tend to underestimate risk and develop too ambitious plans that commonly violate limitations in worst-case realizations. The semantic value of uncertainty is lost by centroid/defuzzification techniques. The average of possibility and necessity measures (Cr (·) = (Pos (·) + Nec (·))/2) is the credibility (pessimistic) measure, which was first proposed by Liu and Liu (2002)^46^ and subsequently used in production contexts by Roshanaei et al. (2013)^47^ and Rastgar et al. (2023)^48^. It ensures that constraints are satisfied with at least 50% credibility while maintaining risk aversion. This is particularly useful for industrial managers that value practicality and cost management over excessive optimism. Thus, the pessimistic (credibility-constrained) trapezoidal fuzzy programming approach is used in this study.

### Defuzzification structure

Considering the uncertainty of demand parameters and processing time, a pessimistic fuzzy programming model is proposed to control the uncertain parameters as follows. The main reason for considering the problem parameters as fuzzy is the lack of access to historical data and incomplete information. Also, the trapezoidal fuzzy programming method is considered to cover the weaknesses of the triangular fuzzy model: Consider the following linear mathematical programming model with the fuzzy parameter of processing time:

20 $$Min\,Z = {\text{ c}}^{t} x$$

21 $$\begin{aligned}&\mathrm{s.t.:}\\ & x \in N(\tilde{A},\tilde{B}) \, = \, \{ x \in R^{n} |a_{ij} x \ge \tilde{p}_{ji} \} ,\;\;\;i \in m,\;jx \ge \, 0 \end{aligned}$$

Where *A* = [*aij*]_*m*×*n*_ , *p̃*_ij_ = (*p̃*_1_,*p̃*_2_,…,*p̃*_*n*_)*t* , *c* = (*c*_1_,*c*_2_,…,*c*_*n*_) is the parameter used in the objective function of the problem, the coefficient vector and the parameter on the right side of the constraint (processing time/demand value). The probability distribution function of the fuzzy parameter of processing time and demand is assumed based on the properties of fuzzy numbers. Finally, *x* = (*x*_1_, *x*_2_, …, *x*_*n*_) represents the decision vector. For the feasibility and optimization of the problem presented in the above model, it is necessary to control the uncertain parameter presented in the objective function and constraint. Therefore, assuming the parameter α as the minimum degree of feasibility of the constraints, the controlled model is as follows:

22 $$Min\,Z = {\text{ c}}^{t} x$$

23 $$\begin{aligned}&\text{s.t.: }\\ &a_{ij} x \ge \, ({1} - \alpha )E_{{1}}^{pji} + \alpha E_{{2}}^{pji} ,\;\;\;i \in m,j \end{aligned}$$

24 $$x \ge \, 0,\alpha \in \left[ {0,{1}} \right]$$

In the above equation, *E*_1_^*bi*^, *E*_2_^*bi*^ is the expected value of the fuzzy number of the processing time and demand parameter used on the right side of the constraint, which is calculated as follows:

25 $${E}_{1}^{{P}_{ij}}=\frac{{P}_{ij}^{1} + {P}_{ij}^{2}}{2}$$

26 $${E}_{2}^{{P}_{ij}}=\frac{{P}_{ij}^{3} + {P}_{ij}^{4}}{2}$$

**Fig. 1:**
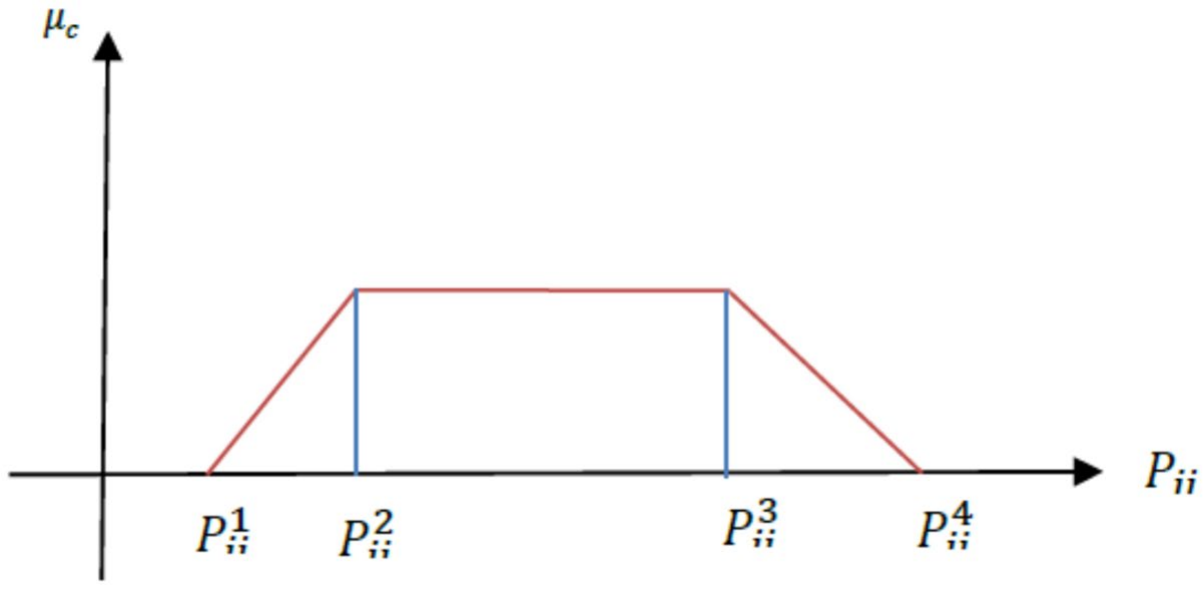
Trapezoidal probability distribution of fuzzy parameter.

In this research, the fuzzy parameters are considered as trapezoidal fuzzy numbers as shown in Fig. 1.

### Numerical illustration of trapezoidal defuzzification

Because historical data for demand and human/robot processing times are either few or unavailable in the investigated low-volume/customized human–robot cells, epistemic uncertainty is modelled using trapezoidal fuzzy numbers. The four values $\widetilde{\xi }$ = (${\xi }_{1}, {\xi }_{2}, {\xi }_{3}, {\xi }_{4}$) define a general trapezoidal fuzzy parameter $\widetilde{\xi }$. The most pessimistic, most plausible lower, most plausible upper, and most optimistic values are represented by ${\xi }_{1}$ ≤ ${\xi }_{2}$ ≤ ${\xi }_{3}$≤ ${\xi }_{4}$, respectively (see Fig. 1).

In accordance with Liu and Liu (2002), the risk-averse criterion is the credibility measure Cr {$\xi$ ≤ r}. For any constraint containing a fuzzy parameter on the right-hand side (e.g., demand τ̃ _pt_ or processing time $\widetilde{\updelta }$ pwh, the pessimistic credibility-constrained equivalent requires that the constraint holds with credibility of at least α (α ≥ 0.5):

Cr {fuzzy expression involving $\widetilde{\xi }$} ≥ α.

For a trapezoidal fuzzy number $\widetilde{\xi }$ = (${\xi }_{1}, {\xi }_{2}, {\xi }_{3}, {\xi }_{4}$), the credibility inversion yields the following deterministic equivalent (Liu & Liu, 2002; Pishvaee & Torabi, 2010):

Cr {$$\widetilde{\xi }$$ ≤ r} ≥ α ⇔ r ≥ (1 − α) $${\widetilde{\xi } }^{3}$$+ α $${\widetilde{\xi } }^{4}$$ when α > 0.5

Cr {$$\widetilde{\xi }$$ ≥ r} ≥ α ⇔ r ≤ (1 − α) $${\widetilde{\xi } }^{2}$$+ α $${\widetilde{\xi } }^{1}$$ when α > 0.5

Concrete numerical example.

Consider product p = 1 processed at workstation w = 1 in period t = 1. Let the fuzzy processing time when performed by a robot (h = 1) be:

$${\widetilde{\updelta }}_{(\mathrm{1,1},1)}== (28, 32, 38, 45)\text{ hours}$$

and the fuzzy demand in period t = 1 be:

$${\widetilde{\uptau }}_{(\mathrm{1,1})}= (180, 200, 220, 250)\text{ units}.$$

Assume the decision-maker chooses a minimum credibility level α = 0.8 (highly risk-averse).

Processing-time constraint (completion time definition, original Eqs. 11–14):

The original fuzzy constraint contains $\widetilde{\updelta }$ pwh on the right-hand side and has the form “ ≤ fuzzy parameter”. Its credibility-constrained crisp equivalent (α = 0.8) is:

C _(1,1)_ ≥ X _(1,1)_ · [(1 − 0.8) ·38 + 0.8·45].

C _(1,1)_ ≥ X _(1,1)_ · (0.2·38 + 0.8·45).

C _(1,1)_ ≥ X _(1,1)_ · 43.6

 → The fuzzy processing time is replaced by the crisp value 43.6 h.

2. Demand/inventory balance constraint (original Eqs. 6 and 9):

These constraints contain demand on the right-hand side in “ ≤ τ̃_(pt)_” form. The crisp equivalent (α = 0.8) becomes:

I _(1,1)_ + Y _(1,0)_ – Y _(1,1)_ ≤ (1 − 0.8) ·220 + 0.8·250.

I _(1,1)_ + Y _(1,0)_ – Y _(1,1)_ ≤ 244.

Therefore, the pessimistic α = 0.8 forces the model to respect a processing time of 43.6 h and a demand bound of 244 units values that lie toward the pessimistic side of the membership function instead of using the core value (32–38 h) or the optimistic centroid. This ensures that the schedule remains feasible with at least 80% credibility even in unfavorable realizations.

This credibility-based rule with α = 0.8 is used to translate all fuzzy processing times $\widetilde{\updelta }$ pwh and demands τ̃_pt_ in the real industrial case and the solved numerical examples (unless otherwise mentioned in sensitivity studies). This transformation gives the final mixed-integer linear programming model that is solved by the ε-constraint technique and the meta-heuristics.

Finally, the deterministic and controlled model of the problem is as follows.

27 $$Max\,NPV\, = \,\mathop \sum \limits_{t} \frac{{\mathop \sum \nolimits_{p} \begin{array}{*{20}c} {\left( {kptXpt - \left( {\vartheta ptXpt + \alpha ptYpt + \mu ptSpt} \right)} \right) - } \\ {B_{{t \pi_{t} }} - \mathop \sum \nolimits_{p} \mathop \sum \nolimits_{w} \sigma pwRpwh^{1} t} \\ \end{array} }}{{\left( {1 + i} \right)^{t} }}$$

28 $$Min\,Cmax = U_{max}$$

29 $$\mathop {Max\,ELtime\, = \,\sum }\limits_{p} \mathop \sum \limits_{w} \left( {E_{{{\mathrm{pw}}}} + { }T_{{{\mathrm{pw}}}} } \right)$$

30 $$\begin{aligned}&\mathrm{s.t:}\\ &\sum_{p}\sum_{h}\sum_{t^{\prime}\le t}{\beta }_{pt^{\prime}t} {I}_{pt^{\prime}} {R}_{pwht} \left({\alpha }\left(\frac{{\delta }_{pwh}^{3} + {\delta }_{pwh}^{4}}{2}\right)+\left(1- {\alpha }\right)\left(\frac{{\delta }_{pwh}^{1} + {\delta }_{pwh}^{2}}{2}\right)\right) {\widetilde{\updelta }}_{pwh \le {\upzeta }_{wt} {{\varphi }}_{wt} }, \forall w,t\end{aligned}$$

31 $$\mathop {X_{pt}} = \sum \limits_{t^{\prime} \le t} \beta_{pt^{\prime}t} I_{pt^{\prime}} , \forall p,t$$

32 $$Y_{pt} {-}S_{pt} = X_{pt} {-}({\alpha }\left( {\frac{{\tau_{pt}^{3} + \tau_{pt}^{4} }}{2}} \right) + \left( {1 - {\upalpha }} \right)\left( {\frac{{\tau_{pt}^{1} + \tau_{pt}^{2} }}{2}} \right) + Y_{pt - 1} - S_{pt - 1} , \;\;\;\forall p,t$$

33 $$\sum_{p}\sum_{w}\sum_{t^{\prime}\le t}{\beta }_{pt^{\prime}t} {I}_{pt^{\prime}} {R}_{pwht} \left({\alpha }\left(\frac{{\delta }_{pwh}^{3} + {\delta }_{pwh}^{4}}{2}\right)+\left(1- {\alpha }\right)\left(\frac{{\delta }_{pwh}^{1} + {\delta }_{pwh}^{2}}{2}\right)\right) \le {\theta }_{t} {B}_{t}, \forall t,h=1$$

34 $$\sum_{h}{R}_{pwht}=1, \forall p,t,w\in {w}_{p}$$

35 $$\sum{I}_{pt} \le \sum_{t}\left({\alpha }\left(\frac{{\tau }_{pt}^{3} + {\tau }_{pt}^{4}}{2}\right)+\left(1- {\alpha }\right)\left(\frac{{\tau }_{pt}^{1} + {\tau }_{pt}^{2}}{2}\right)\right),\forall \mathrm{p}$$

36 $${B}_{t}^{min} \le {B}_{t} \le {B}_{t}^{max} , \forall \mathrm{t}$$

37 $${C}_{pw} \ge \sum_{h}{\varpi }_{pwh} \left({\alpha }\left(\frac{{\delta }_{pwh}^{3} + {\delta }_{pwh}^{4}}{2}\right)+\left(1- {\alpha }\right)\left(\frac{{\delta }_{pwh}^{1} + {\delta }_{pwh}^{2}}{2}\right)\right), \forall p,w\in {w}_{p}^{init}$$

38 $${C}_{pw} \ge {C}_{pw-1}+ \sum_{h}{\varpi }_{pwh} \left({\alpha }\left(\frac{{\delta }_{pwh}^{3} + {\delta }_{pwh}^{4}}{2}\right)+\left(1- {\alpha }\right)\left(\frac{{\delta }_{pwh}^{1} + {\delta }_{pwh}^{2}}{2}\right)\right), \forall p,w\notin {w}_{p}^{init}$$

39 $${C}_{pw} \ge {C}_{p^{\prime}w-1}+ \sum_{h}{\varpi }_{pwh} \left({\alpha }\left(\frac{{\delta }_{pwh}^{3} + {\delta }_{pwh}^{4}}{2}\right)+\left(1- {\alpha }\right)\left(\frac{{\delta }_{pwh}^{1} + {\delta }_{pwh}^{2}}{2}\right)\right)-K \left(1- {H}_{p{p}^{\prime}w} \right), \forall w, {p}^{\prime}>p$$

40 $${C}_{p^{\prime}w} \ge {C}_{pw}+ \sum_{h}{\varpi }_{p^{\prime}wh} \left({\alpha }\left(\frac{{\delta }_{p^{\prime}wh}^{3} + {\delta }_{p^{\prime}wh}^{4}}{2}\right)+\left(1- {\alpha }\right)\left(\frac{{\delta }_{p^{\prime}wh}^{1} + {\delta }_{p^{\prime}wh}^{2}}{2}\right)\right)-K \left({H}_{p{p}^{\prime}w} \right), \forall w, {p}^{\prime}>p$$

41 $${U}_{max} \ge {C}_{pw} , \forall p,w\in {w}_{p}^{last}$$

42 $$E_{pw} = max[D_{pw} - C_{pw} ,0],\;\;\;\forall p,w \in w_{p}^{last}$$

43 $${\mathrm{T}}pw = max[{\mathrm{C}}_{pw} - {\mathrm{D}}_{pw} ,0],\;\;\;\forall p,w \in w_{p}^{last}$$

44 $$I_{pt} ,X_{pt} ,Y_{pt} ,S_{pt} ,B_{t} ,C_{mw} ,U_{max} ,E_{pw} ,T_{pw} \ge \, 0$$

45 $$R_{pwht} , \, {\rm H}_{{p}p^\prime w} \in \, \left\{ {0,{1}} \right\}$$

### Clarification of shortage penalty mechanisms and explicit cost-function formulations

Shortages are allowed and treated as backorders with a per-unit per-period penalty cost ${\mu }_{pt}$. Through the term $\sum_{p,t}{\mu }_{pt}{S}_{pt}$ in the first objective function, the associated penalty directly lowers the net present value. This makes the shortage penalty’s function and formulation completely transparent. The shortage quantity ${S}_{pt} \ge 0$ is calculated from the inventory balance (Eq. 32).

### Validation of the universal workstation assumption

We assume universal workstations, as is common in hybrid flow-shop and flexible job-shop literature with human–robot collaboration^12,13^. Any product can be processed at any workstation by either a robot (h = 1) or a human operator (h = 0), with processing time depending on both resource type and product. This is representative of contemporary collaborative cells with multifunctional robots and cross-trained human operators.

By reviewing the literature on the subject such as Roshanaei et al. (2013)^47^, Loukil et al. (2005)^49^ and Rastgar et al. (2023)^48^, it can be stated that production planning models as well as human–robot communication scheduling are categorized as Np-Hard problems. This indicates that metaheuristic or heuristic algorithms should be used to solve numerical examples in larger sizes. Therefore, in this paper, the epsilon constraint method has been used to validate the mathematical model and NSGA-II, MOPSO and MOWOA have been used to solve numerical examples with different sizes. This section of the paper briefly introduces the solution methods and also designs the initial solution for metaheuristic algorithms. At the end of this section, the parameter setting of metaheuristic algorithms has also been done.

#### Designing the initial solution

Before introducing metaheuristic algorithms, the initial solution design of the algorithms is discussed. The main basis of searching the problem space in metaheuristic algorithms is the design of the initial solution. This solution, which is a set of random numbers, allows the algorithm to search the feasible problem space and show the best solutions from the search. To explain the initial solution, a hypothetical problem consisting of 4 workstations, 3 types of products for 3 time periods is considered. The purpose of designing the initial solution is to first assign the type of resource (human/robot) to produce each type of product at each workstation and then determine the amount of product production at each workstation.

**Fig. 2:**
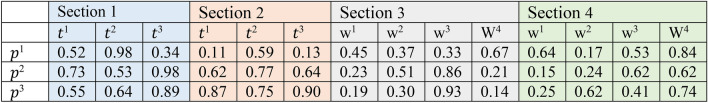
Initial solution to the production planning and scheduling problem of human–robot communication.

Therefore, to assign values ​​to the decision variables of the problem, the initial solution is considered as a vector of length 2 (|*P*|.|*T*| +|P|.|W|) consisting of random numbers between 0 and 1. The main reason for creating random data between 0 and 1 in the initial solution is the continuous search of metaheuristic algorithms. Figure 2 shows the initial solution for the hypothetical problem. In this figure, the initial solution has been transformed from a vector to a (|*P*|× (2|*T*|+ 2|*W*|) matrix, consisting of 4 different sections.

In Fig. 2, section "Introduction" is related to “percentage of product quantity supplied in each period”, Section "Research background" is related to “percentage of product stored at the end of the time period”, section "Research methodology" is related to “human–robot assignment to each station”, and Section "Data analysis and research findings" is related to “job processing sequence”. Since in section "Research methodology", the human–robot assignment to each station must be a number 0 or 1 and in Section "Data analysis and research findings", the job processing sequence must be a permutation of integers, the following correction mechanism is applied to these two sections.

**Fig. 3:**
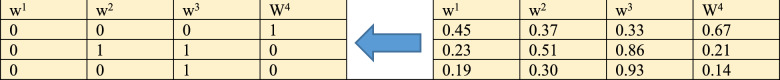
Correction mechanism on Section "Research methodology" of the initial solution.

Section "Research methodology": For each workstation, if the random data is less than 0.5, it takes the value 0 (human) and otherwise takes the value 1 (robot). Figure 3 shows the correction mechanism for Section "Research methodology".

**Fig. 4:**
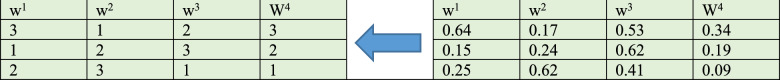
Correction mechanism on Section "Data analysis and research findings" of the initial solution.

Section "Data analysis and research findings": For each workstation, the smallest random number takes the value 1, the next smallest number takes the value 2, etc. Hence, the correction mechanism for Section "Data analysis and research findings" will be as shown in Fig. 4.

**Fig. 5:**
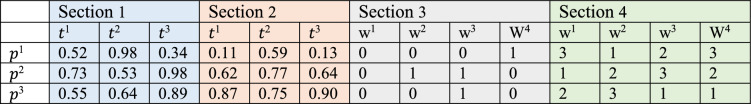
Modified initial solution to the production planning and scheduling problem of human–robot communication.

Considering the modification mechanisms used to convert a s of the continuous space of the initial solution to a discrete space, Fig. 5 shows the modified initial solution of the problem.

Although it is technically possible to generate the assignment section directly as binary 0/1 values and the sequencing section directly as random integer permutations, a continuous representation with subsequent correction was deliberately chosen for the following reasons that significantly improve algorithmic performance in practice:

- Compatibility with all three meta-heuristics: MOPSO and MOWOA are essentially intended for continuous search spaces. By using a completely continuous encoding, NSGA-II, MOPSO, and MOWOA may all use the same solution representation and decoding process without any changes, making implementation easier and guaranteeing fair comparison.
- Superior exploratory capabilities of continuous operators: Continuous crossover (e.g., simulated binary crossover, arithmetic crossover) and mutation operators generate offspring that are modest, regulated perturbations of the parents, leading to smoother movement in the search space and greater preservation of useful building blocks. In contrast, directly applying discrete permutation operators (swap, insertion, etc.) or binary bit-flip mutation typically causes huge disruptive changes, especially in early generations, which slows convergence and decreases solution quality in large-scale problems.
- Empirical superiority: Initial tests performed during the development of the algorithm indicated that continuous encoding with rounding and ranking correction consistently achieved 8–14% higher hypervolume and produced 15–20% more non-dominated solutions compared to fully discrete encoding. Notably, this improvement required nearly the same computation time, as the correction step, with complexity O(|P|·|W|), is negligible relative to the time spent on objective function evaluations. This approach of mapping continuous variables to discrete ones, commonly referred to as "random-keys" for permutations and rounding for binary variables, is a well-established and effective method in the meta-heuristic literature, particularly for tackling scheduling and assignment problems^50^.

To decode this initial solution, the following steps are performed:

**Step 1:** Section "Introduction" represents the “percentage of the quantity of the product supplied in each period.” Hence, the quantity of products supplied (I_*pt*_) can be calculated according to Eq. (35) in proportion to the uncertain demand in each time period (τ̃_*pt*_).

**Step 2:** Based on step 1, the effective amount of product produced (*X*_*pt*_) is calculated based on the loading rate (β_*pt*′*t*_) according to Eq. (31).

**Step 3:** Sect. 2 represents the “percentage of product stored at the end of the time period.” Hence, based on Step 2, section of the effective amount of product produced (*X*_*pt*_) is considered as the amount of product stored at the end of each time period (*Y*_*pt*_).

**Step 4:** Based on the variables obtained in steps 1 to 3 and based on Eq. (32), the amount of shortage (*S*_*pt*_) or product reserve (*Y*_*pt*_) is calculated in time periods.

**Step 5:** Section "Research methodology" represents the "robot-human assignment to each station". If this value is equal to 0, it means that a human is assigned to produce that product at the workstation, and if this value is equal to 1, it means that a robot is assigned to produce that product at the workstation (*R*_*pw*ℎ*t*_).

**Fig. 6:**
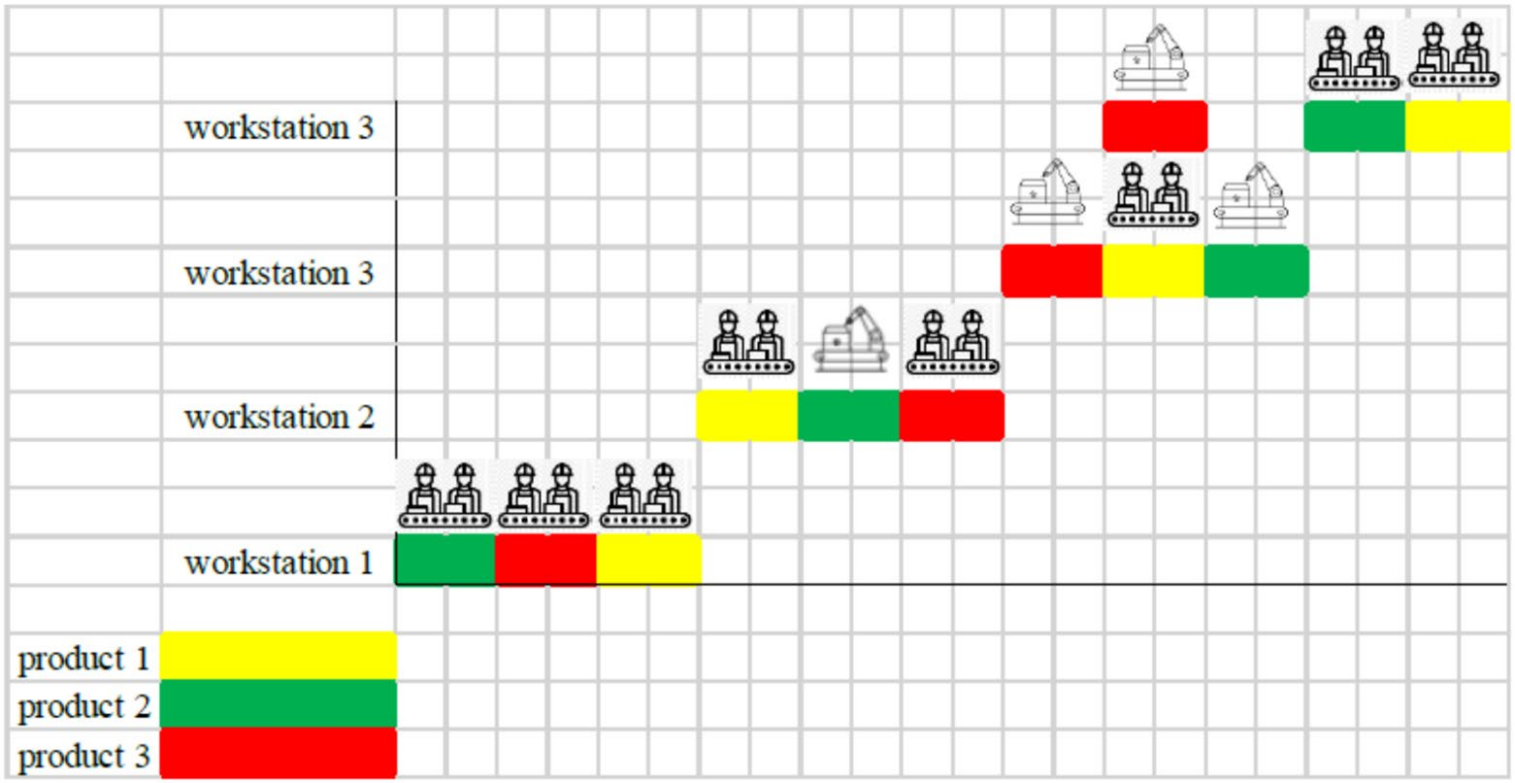
Scheduling of human–robot communication in the hypothetical problem.

**Step 6:** Section "Data analysis and research findings" shows the "job processing sequence at each station". For example, at workstation number (1), product 2 will be processed with priority 1, product 3 with priority 2, and then product 1 with priority 3. Figure 6 shows this sequence of operations for the hypothetical problem.

**Step 7:** The processing schedule for each product at each station will be such that the work at the previous station is fully processed and the human–robot at the next station is idle (Η_*pp*′*w*_).

**Step 8:** The total number of robots in each time period is calculated (*B*_*t*_).

**Step 9:** Product completion time (*C*_*pw*_), product production completion time at the last station (*U*_*max*_), early delivery time (*E*_*pw*_), and late delivery time (*T*_*pw*_) are calculated according to Eqs. (37) to (43).

**Step 10:** Other constraints of the problem are examined based on the decision variables, and if a reasonable problem space is not created, the penalty function is used to deal with this issue (relations 30, 33, 36).

**Step 11:** The values ​​of the objective functions of the problem are calculated.

After designing the initial solution, a brief description of the NSGA-II, MOPSO, and MOWOA algorithms is given below. The initial solution provided for all the proposed algorithms is the same.

### Algorithm NSGA-II

**Fig. 7:**
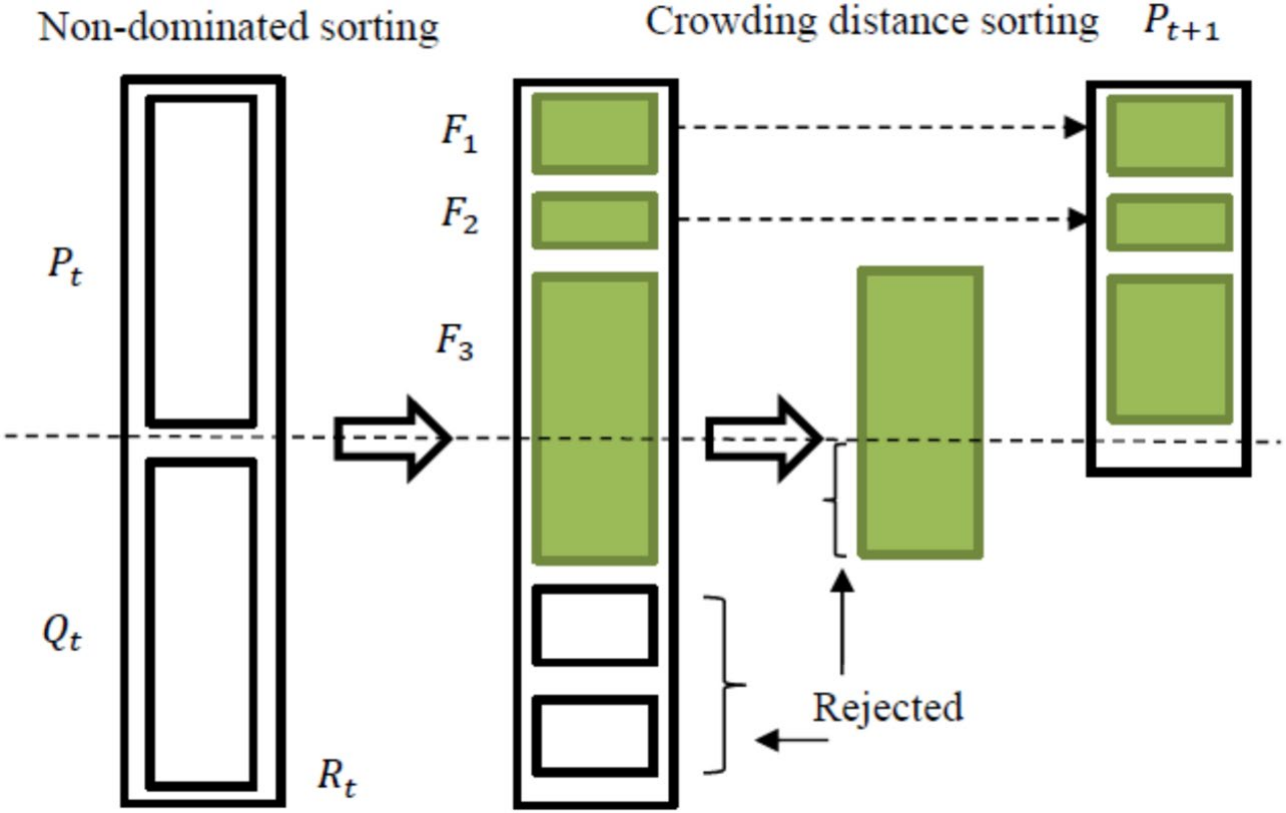
The main structure of the NSGA-II algorithm.

This algorithm was proposed by Deb et al. (2002)^51^ and addresses the weaknesses of classical optimization methods such as computational complexity, non-elitism, and the need to specify a sharing parameter. The NSGA-II algorithm uses elitism to create a Pareto-optimal front. When applying genetic algorithm operators, the elitism method retains the good members of the previous generation to produce the new generation, which in addition to accelerating convergence to the optimal response, also makes the search process more efficient. This algorithm, with a selective function while respecting the elitism principle, creates a new population from the combination of the parent and child populations by applying mutation and combination operators, and selects the best responses according to their fitness and dispersion. In fact, in this algorithm, the responses are first ranked based on the non-dominated response, then sorted based on the crowding distance^18^. In the NSGA-II algorithm, the parameters of maximum number of iterations, population number, crossover percentage, and mutation percentage are determined by trial and error. The main structure of the algorithm is shown in Fig. 7:

The steps for implementing the NSGA-II algorithm are as follows:

1. Create a random initial population P_0_ of size N.
2. Sorting the initial population based on non-dominated answers.
3. Ranking each answer based on the most popular answers.
4. Applying selection, combination, and mutation operators on P_0_ to create a population of offspring Q_0_ of size N.
5. After the first generation is produced, which includes the chromosomes of the parents and children, the new generation is produced as follows:

- Combining the chromosomes of the P_0_ parents and the Q_0_ children and producing the R_t_ generation of size 2N.
- Sorting the R_t_ generation based on the non-dominated classification method and identifying and classifying non-dominated fronts (F_1_, F_2_ …).
- Generation of parent generation with size N for the next iteration (P_t + 1_) using non-dominated fronts. In this step, considering the number of chromosomes required for the parent generation (N), first the number of chromosomes of the first front is selected for the parent generation and if this number does not match the total number required for the parent generation, it is taken from fronts 2, 3, etc. to reach the number (N). If we want to select a limited number of chromosomes in a front, chromosomes that have a greater density distance are selected.
- Applying selection, combination, and mutation operations on the new parent generation (P_t + 1_) and producing a generation of children (Q_t + 1_) of size N.
- Repeat from step 5 until the stopping condition is reached.

**Fig. 8:**
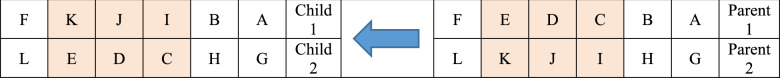
How to perform the single-point combination operator.

**Fig. 9:**
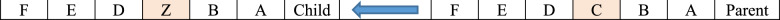
How to perform the transition jump operator.

In this algorithm, the combination and mutation operators are used to generate the new generation. Figures 8 and 9 present the implementation mechanism of the two-point combination and single-point mutation operators.

The most important operator in the NSGA-II algorithm is the combination operator. Combination is the process in which old generations of chromosomes are mixed and combined to form new generations of chromosomes. The pairs that were considered as parents in the selection section exchange their genes with each other in this section and create new members. Combination in the genetic algorithm causes the loss of dispersion or genetic diversity of the population because it allows good genes to find each other. As mentioned above, the way to represent the answers in this paper is with permutation numbers and there are several corresponding combination methods in the literature. In this paper, two-point combination is used to combine chromosomes. Figure 8 shows how combination is applied to the problem chromosomes.

The mutation operator is another operator that generates other possible solutions. In the NSGA-II algorithm, after a member is created in a new population, each gene in it is mutated with a certain mutation probability. In mutation, a gene may be removed from the population gene set or a gene that has not yet existed in the population may be added to it. Mutating a gene means changing that gene, and depending on the type of encoding, different methods have been developed in the literature. In this paper, the transition mutation is used for the mutation operator between chromosomes of the order package merging and pickup routing problem. Figure 9 shows how to apply and transform this chromosome into a valid solution.

### Algorithm MOPSO

This algorithm is a generalized particle swarm optimization algorithm that is used to solve multi-objective problems. In the MOPSO algorithm, there is a concept called archive or storage, in which a set of unsuccessful responses that approximate the Pareto front is archived. The particle swarm optimization algorithm operates based on collective intelligence. In this algorithm, each particle has specific characteristics of position, speed, and direction of movement. Movement is important in this algorithm because it exchanges information and creates convergence between particles. Each particle has three sources of information for movement, including the behavior that the particle has shown before and tries to repeat its last activity, the best location it has experienced in the search space, and the best location that all particles have experienced in the search space. In the MOPSO algorithm, the parameters of the maximum number of iterations, the population number, the number of solutions in the reservoir, and the mutation rate are determined by trial and error. The equations describing the behavior of particles are as follows:

46 $$v_{i} (t + {1}) \, = w \times v_{i} (t) \, + c_{{1}} r_{{1}} (xbest_{i} (t) \, - x_{i} (t)) \, + c_{{2}} r_{{2}} (xgbest(t) \, - x_{i} (t))$$

47 $$x_{i} (t + {1}) \, = x_{i} (t) \, + v_{i} (t + {1})$$

*x*_*i*_ (*t*) represents the position of particle i at time t, *v*(*t*) represents the velocity of particle i at time t, be represents the previous best position of particle i, and *xbest*_*i*_(*t*) represents the position of the best particle in the entire space, r_1_ and r_2_ are random numbers between 0 and 1, c_1_ and c_2_ are the acceleration coefficients, and w represents the inertia coefficient.

The steps for implementing the MOPSO algorithm are as follows:

1. Creating the initial population.
2. Separating non-dominant members of the population and storing them in a reservoir.
3. Tabulation of the discovered target space (meaning that, considering the minimum and maximum values of the obtained target functions, each of these intervals is divided into 5 to 10 equal sections based on the need).
4. Selecting a leader from the pool for each particle and moving it.
5. Updating the best personal memory of every particle.
6. Add non-dominated members of the current population to the pool.
7. Delete the storage members.
8. Due to lack of memory in the repository, we need to reduce their number. If the number of storage members exceeds the specified capacity, we remove the excess members. Cells with larger populations are prioritized for removal because it is more important for us to maintain diverse responses.
9. If the termination conditions are not met, we return to step 3, otherwise we proceed to the end step.

### MOWOA algorithm

The Whale Optimization Algorithm is a swarm intelligence optimization algorithm inspired by the unique hunting method of sperm whales, called the bubble attack approach. Schools of krill, or small fish, are the preferred food of humpback whales. When humpback whales are hunting, they exhibit two types of bubble network-related behaviors, one of which is the upward spiral behavior. In this behavior, they dive down and begin to create bubbles in a spiral shape around the prey and swim to the surface. The second behavior is called the double ring, which has three stages: the coral ring, the tail lobe, and the grab ring. The logic of the Whale Optimization Algorithm is to mimic the behavior of humpback whales.

To formulate the MOWOA algorithm, the coefficient vector $\overrightarrow{A}$ and $\overrightarrow{C}$ is calculated as follows:

48 $$\vec{A}~ = {\text{ 2}}.\vec{a}.\vec{r} - \vec{a}$$

49 $$\vec{C} = { 2}.\vec{r}$$

Where *a⃗* is a vector that linearly decreases from two to zero in iterations and *w* is considered as a random number in [0, 1].

- If *w* < 0.5 and |*A⃗*|≤ 1, the prey encirclement phase begins. Humpback whales can detect prey locations and surround them. The current best solution for the target prey is at or near the optimal one. After defining the best search solution, other search solutions try to update their positions relative to it. This behavior is represented by the following equations:
  50 $$\vec{D} = \left| {\vec{C}~ \times ~\overrightarrow {{P_{*}^{t} ~ - ~P_{l}^{t} }} } \right|$$
  51 $$\overrightarrow{{P}_{i}^{t +1}} = \overrightarrow{{P}_{l}^{t}} - \overrightarrow{A} \times \overrightarrow{D}$$
  where *t* represents the current iteration, $\overrightarrow{{P}_{*}^{t}}$ is the position vector of the best solution obtained so far, $\overrightarrow{{P}_{l}^{t}}$ is the position vector, and | | is the absolute value. If a better solution exists, $\overrightarrow{{P}_{*}^{t}}$ should be updated at each iteration.
- If *w* ≥ 0.5 and |*A⃗*|≤ 1, the bubble attack phase begins. Inside the contracting chamber, humpbacks follow spiral paths towards their prey, a method known as bubble network search. Therefore, the whale optimization algorithm calculates the distance between the whale and its prey when it uses the spiral method to update its position. The mathematical formula for simulating the spiral motion of a humpback whale is as follows:
  52 $$\overrightarrow{{P}_{i}^{t +1}} = \overrightarrow{{D}^{\prime}}. {e}^{bl}.\mathrm{cos}(2\pi l)+ \overrightarrow{{P}_{*}^{t}}$$
  where $\overrightarrow{D}= \left|\overrightarrow{{P}_{*}^{t}}- \overrightarrow{{P}_{l}^{t}}\right|$ represents the distance of the *i*th wall to the best solution obtained so far, *b* is a constant to define the shape of the logarithmic spiral, and *l* is a random number in [− 1.1].
- If *w* < 0.5 and |*A⃗*|> 1, the random search phase for prey begins. In the process of hunting prey, whales must locate their prey. Once the location is determined, whales can surround their prey. The current best search agent is considered as the target prey. All whales update their positions with the tendency to approach the prey. This procedure is repeated until a predetermined condition is reached. The computational model of individuals to update their positions is expressed by the mathematical formula as follows:
  53 $$\vec{D} = \left| {\vec{C} \times \overrightarrow {{P_{rand} }} - \overrightarrow {{P_{l}^{t} }} } \right|$$
  54 $$\overrightarrow{{P}_{i}^{t +1}} = \overrightarrow{{P}_{rand}}- \overrightarrow{A} \times \overrightarrow{D}$$
  where $\overrightarrow{{P}_{rand}}$ is the position vector randomly generated in the boundary area, $\overrightarrow{{P}_{l}^{t}}$ is the *i*th position vector of the *t* generation of the search factors, and $\overrightarrow{{P}_{i}^{t +1}}$ is the second position vector of the *t* + 1 of the search factors.

After examining the proposed methods for solving the problem, the parameter setting of metaheuristic algorithms using the Taguchi method has been addressed. The purpose of this work is to adjust the initial parameters of each algorithm in order to increase its efficiency in searching for quality solutions. To derive a unified composite performance index that balances both solution quality and computational cost, a normalized composite metric (Y) is computed for each algorithm i by integrating the five individual performance metrics in the following manner:

55 $$Yi=NPFi+MSIi+SMi-MIDi-CPTi$$

where all five metrics (NPF, MSI, SM, MID, CPT) are first normalized to the [0,1] interval across the three algorithms using the min–max normalization technique:

Metric norm = Metric – MetricminMetricmax − Metricmin.

This normalization removes the effects of varying scales and units across the metrics. Within the composite index (Eq. 55):

- NPF (Number of Pareto Solutions), MSI (Maximum Spread Index), and SM (Spacing Metric) are metrics used to assess the quality and diversity of the Pareto front. Since higher values for these indicators signify better performance, they are combined accordingly.
- MID (Mean Ideal Distance) and CPT (Computation Time) are indicators that are minimized, meaning lower values are preferable; therefore, they are subtracted.

The highest Y value signifies the algorithm that most effectively harmonizes the factors of quantity, uniformity, spread, proximity to the ideal point, and computational efficiency among the obtained non-dominated solutions. Based on the averaged results from the 15 test cases (refer to Table 9), MOWOA attains the highest composite score (NCMMOWOA = 2.37), outperforming NSGA-II (1.68) and MOPSO (1.12). This outcome highlights MOWOA’s overall superiority as judged by the integrated numerical criterion outlined in Eq. (55).

In this method, the initial parameters are first set based on the proposed levels according to the Taguchi experiments and their RPD (Relative Percentage Deviation) values ​​are obtained according to the following relations.

56 $${RPD}_{i}= \frac{{Y}_{i}- {Y}^{*}}{{Y}^{*}}$$

### Streamlining metaheuristic descriptions and full tabular documentation of parameters

In the above relations, ${Y}_{i}$ is the response of each Taguchi experiment and ${Y}^{*}$ is the best response among all experiments. Also, the number of efficient solutions is shown by NPF (Number of Pareto Front), the most spread by MSI (Maximum Spread Index), the metric distance by SM (Space Metric), the distance from the ideal point by MID (Mean of Ideal Deviations) and the solution time by CPT (Computation Time).

After determining the RPD value, the optimal levels of the parameters are obtained. In Table 2, the proposed and optimal levels obtained in parameter tuning of meta-heuristic algorithms are presented.

**Table 2:** Tuned parameters of the metaheuristic algorithms after Taguchi calibration.

Algorithm	Parameter	Suggested levels			Selected (optimal) value
		Level 1	Level 2	Level 3	
NSGA-II	Population size (Npop)	100	150	200	200
	Maximum iterations (MaxIt)	200	300	400	400
	Crossover percentage (Pc)	0.7	0.8	0.9	0.8
	Mutation percentage (Pm)	0.1	0.2	0.3	0.2
	Mutation rate per gene	0.02	0.05	0.1	0.05
	Selection method	Tournament (size = 3) – fixed	Tournament (size = 3)		
MOPSO	Population size (Npop)	100	150	200	200
	Repository (archive) size	50	100	150	100
	Maximum iterations (MaxIt)	200	300	400	400
	Inertia weight (w)	0.4	0.7	1.0	0.7
	Personal learning coefficient (c1)	1.0	1.5	2.0	2.0
	Global learning coefficient (c2)	1.5	2.0	2.5	2.0
	Number of grids per dimension	5	10	15	10
	Mutation rate	0.1	0.2	0.3	0.1
MOWOA	Population size (Npop)	100	150	200	200
	Maximum iterations (MaxIt)	200	300	400	400
	Spiral shape constant (b)	0.5	1.0	1.5	1.0
	Leader selection pressure (for archive)	-	-	-	Adaptive (repository size = 100)
	Archive size (non-dominated solutions)	50	100	150	100
	Grid inflation parameter (α)	0.1	0.2	0.3	0.1
	Deletion selection pressure (p)	2	4	6	4

All methods employ the same solution representation and decoding procedure described in Section "Research methodology"

Parameter tuning was performed using the Taguchi L27 orthogonal array with the Signal-to-Noise ratio (larger-the-better) based on the combined measure (NPF + MSI – SM – MID – CPT)

The selected (optimal) values given in the last column are the ones utilized for all numerical experiments reported in Section "Data analysis and research findings" and Tables 8, 9 and 10

### Final parameter settings of the meta-heuristic algorithms

To ensure full reproducibility, the final parameter values applied in all computational experiments (following Taguchi calibration) are detailed in Table 3.

**Table 3:** Final parameter values of the three meta-heuristic algorithms.

Parameter	NSGA-II	MOPSO	MOWOA
Population (swarm) size	200	200	200
Archive (repository) size	-	100	100
Maximum number of iterations	1000	1000	1000
Crossover probability (pc)	0.90	-	-
Crossover type	Two-point crossover		
Mutation probability (pm)	0.20	Mutation rate linearly increasing from 0.1 to 0.5	-
Mutation type (NSGA-II)	Swap mutation on sequencing part + bit-flip on assignment part	Random perturbation on continuous vector	-
Inertia weight (w)	-	Decreases linearly from 0.9 to 0.4	Decreases linearly from 2 to 0 (coefficient a)
Personal learning coefficient (c1)	-	2.0	-
Global learning coefficient (c2)	-	2.0	-
Grid divisions for archive	-	10	10
Leader selection pressure	-	Roulette-wheel on least crowded cells	Roulette-wheel on least crowded cells
Spiral shape constant (b)	-	-	1.0
Stopping criterion	Maximum iterations	Maximum iterations	Maximum iterations

The same parameter values were maintained across all 15 test instances and the real-world industrial case. All algorithms were implemented in MATLAB R2024b and executed on a system equipped with an Intel Core i7-12700H processor, 32 GB of RAM, and running Windows 11.

## Data analysis and research findings

The mathematical model presented in this paper is a three-objective model including maximizing the net present value, minimizing the time to complete the last product, and minimizing the sum of the early and late times. Therefore, the epsilon constraint method has been used to validate the mathematical model, and the NSGA-II, MOPSO, and MOWOA algorithms have been used to solve the mathematical model in different sizes. Therefore, 15 numerical examples in different sizes have been considered according to Table 4.

**Table 4:** Size of numerical examples.

Numerical example	Number of products	Number of time periods	Number of workstations	Number of robots
1	3	3	4	4
2	5	3	4	4
3	8	3	6	4
4	10	4	6	5
5	12	4	8	5
6	15	4	8	5
7	18	6	10	6
8	21	6	10	6
9	25	6	12	6
10	28	8	12	7
11	30	8	15	7
12	33	8	15	7
13	36	12	18	8
14	40	12	18	8
15	45	12	20	8

### Performance indicators

To thoroughly evaluate the performance of the three multi-objective meta-heuristic algorithms NSGA-II, MOPSO, and MOWOA five widely recognized performance metrics are utilized. These metrics are derived based on the Pareto fronts generated by each algorithm at the conclusion of the final iteration.

**Number of Pareto Front solutions (NPF).** The cardinality of the final non-dominated set represents its size. A higher NPF suggests that the algorithm has identified a wider range of diverse compromise solutions, providing decision-makers with increased flexibility.

**Maximum Spread Index (MSI).** Measures how well the obtained Pareto front covers the entire true Pareto region. It is computed as

$$\text{MSI }=\sqrt{\frac{1}{m} \sum_{k=1}^{m}{(\frac{{f}_{k}^{max}- {f}_{k}^{min}}{{f}_{k}^{global max}- {f}_{k}^{global min}})}^{2}}$$

where $m = 3$ is the number of objectives, $${f}_{k}^{max}and {f}_{k}^{min}$$ are the maximum and minimum values of objective k in the obtained front, and $${f}_{k}^{global max}- {f}_{k}^{global min}$$ are the global best and worst values found by any algorithm across all runs. A value closer to 1 indicates superior spread.

**Spacing Metric (SM).** Quantifies the uniformity of the distribution of solutions along the front^52^:

$$SM = \sqrt {\frac{1}{{n - 1}}~\mathop \sum \limits_{{i = 1}}^{n} \left( {d_{i} - ~\bar{d}} \right)^{2} } ,\;\;\;\;\;\bar{d} = ~\frac{1}{n}~\mathop \sum \limits_{{i = 1}}^{n} d_{i}$$

Here, ${d}_{i}$ represents the Euclidean distance (in the normalized objective space) between solution i and its closest neighbor. Smaller SM values indicate a more evenly distributed front.

**Mean Ideal Distance (MID).** determines the average separation between the obtained front and the ideal point (0, 0, 0) following the normalization of all objectives to [0,1]:

$${\mathrm{MID}} = \frac{1}{n} \mathop \sum \limits_{i = 1}^{n} \sqrt {f_{i1}^{{norm^{2} }} + } f_{i2}^{{norm^{2} }} + f_{i3}^{{norm^{2} }}$$

Smaller MID values indicate better convergence toward the ideal point.

**Computational Time (CPT).** Average CPU time (in seconds) required for one complete run of the algorithm.

Together, these five measures assess the created Pareto fronts’ quality (convergence), diversity, homogeneity, and computing efficiency. These well-defined indicators serve as the foundation for all following comparisons.

Also, the bounds of the problem parameter bases are considered based on the uniform distribution function according to Table 5.

**Table 5:** Bounds of the problem parameter bases based on the uniform distribution function.

Parameter	Interval limits	Parameter	Interval limits
δ̃_*pw*0_	${\delta }_{pw0}^{1}$~U (2, 5) ${\delta }_{pw0}^{2}$ ~U (5, 7) ${\delta }_{pw0}^{3}$ ~U (7, 10) ${\delta }_{pw0}^{4}$ ~U (10, 12)		
δ̃_*pw*1_	${\delta }_{pw1}^{1}$~U (1, 3) ${\delta }_{pw1}^{2}$ ~U (3, 5) ${\delta }_{pw1}^{3}$ ~U (5, 8) ${\delta }_{pw1}^{4}$ ~U (0, 10)		
τ̃_*pt*_	${\uptau }_{pt}^{1}$~U (10, 20) ${\uptau }_{pt}^{2}$~U (20, 30) ${\uptau }_{pt}^{3}$ ~U (30, 40) ${\uptau }_{pt}^{4}$ ~U (40, 50)		
θ_*t*_	~ U (500, 800)	µ_*pt*_	~ U (6, 120)
φ_*wt*_	~ U (1500, 1800)	π_*t*_	~ U (200, 1000)
*k*_*pt*_	~ U (100, 200)	σ_*pw*_	~ U (50, 150)
ϑ_*pt*_	~ U (10, 12)	${B}_{t}^{min}, {B}_{t}^{max}$	[0, *R*]
α_*pt*_	~ U (6, 8)	*D*_*pw*_	~ U (40, 80)
β_*pt*′*t*_	~ U (0.5, 0.9)	i	20%
ζ_*wt*_	~ U (0.8, 0.9)		

To validate the mathematical model, the epsilon constraint method has been used, and the set of efficient solutions for numerical example number (1) is shown in Table 6. In this analysis, the uncertainty rate value for dealing with demand parameters and processing time is considered to be 0.5.

**Table 6:** Set of efficient solutions for numerical example number 1 using the epsilon constraint method.

Efficient Solutions	Net present value	Maximum processing time	Total time of early and late arrival
1	12,138.88	58.75	33.25
2	12,039.23	57.15	32.84
3	11,948.68	55.39	31.29
4	11,824.30	53.49	30.49
5	11,628.49	51.27	29.75
6	11,427.34	50.06	29.34
7	11,229.64	48.67	29.17
8	11,175.68	48.34	28.46
9	11,040.09	47.93	28.10
10	10,948.31	47.10	27.64
11	10,816.57	45.83	27.39
12	10,773.26	45.37	27.19

The results of Table 6 show that by reducing the maximum processing time of tasks or the total amount of early and late time, the net present value has also decreased. This is due to the use of robots instead of humans to process and produce products at different workstations. Due to the use of robots, the processing and production time of the product has decreased, and on the other hand, the costs of employing robots have increased. With the increase in costs, the net present value has decreased.

**Fig. 10:**
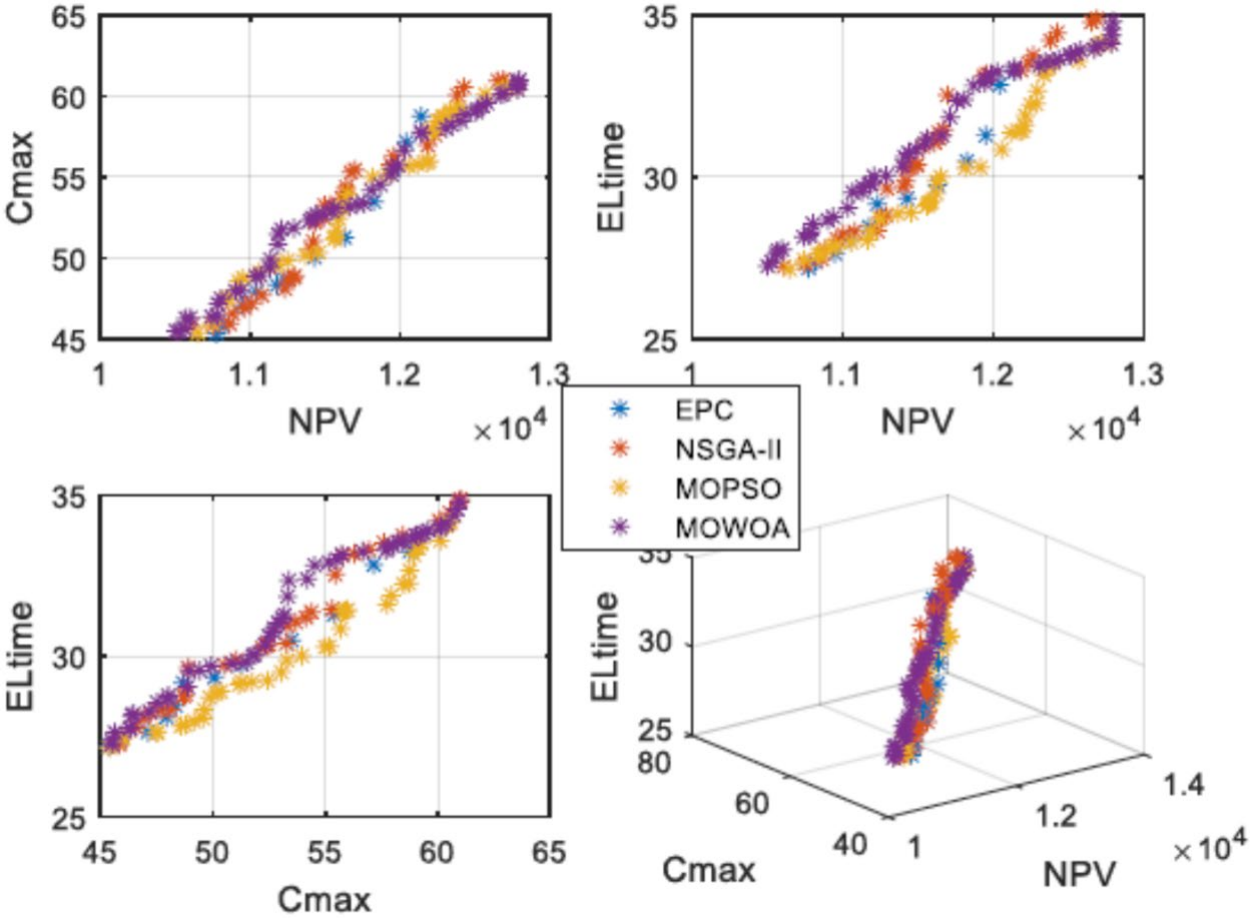
Comparison of the Pareto front obtained from solving numerical example number 1.

In this analysis, 12 efficient solutions were obtained by the epsilon constraint method, and by solving this numerical example with metaheuristic algorithms, 32 efficient solutions were obtained with NSGA-II, 38 efficient solutions with MOPSO, and 50 efficient solutions with MOWOA, respectively. Figure 10 shows the comparison of the Pareto front obtained from solving numerical example number (1) with different solution methods.

**Fig. 11:**
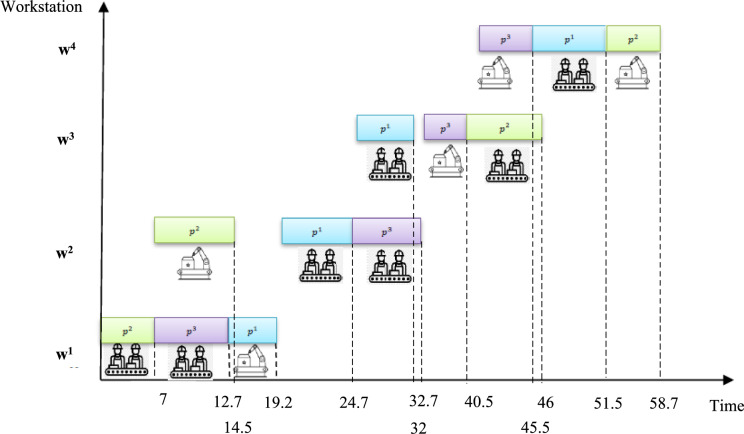
Timing of human–robot communication in the first efficient solution to the problem.

Figure 10 shows that the convergence of the algorithms in solving numerical example number (1) was the same. Next, the analysis of the first efficient solution obtained using the epsilon constraint method is discussed due to the high accuracy of this method. Figure 11 shows the timing of the human–robot communication in the analysis of the first efficient solution of the problem with the epsilon constraint method.

According to Fig. 11, it is observed that the maximum processing time of the last product at the fourth station was 58.75 units. Since the delivery time of the first product is 46, the second product is 73, and the third product is 59, the first product is 5.5 units late, and products 2 and 3 are processed 14.25 and 13.5 units ahead of schedule, respectively. Therefore, the total time of early and late processing is 33.25. Table 7 also shows the amount of production, shortage, and stock of the product in each time period for the first efficient solution.

**Table 7:** Production planning in the first efficient solution of the problem with the epsilon constraint method.

Product	Time period	Demand	Effective amount produced	Deficiency	Inventory
1	1	28.25	11.45	16.80	0
	2	30.75	30.56	16.99	0
	3	31.50	52.30	0	3.81
2	1	31.25	6.15	25.10	0
	2	28.25	19.03	34.30	0
	3	27.50	33.71	28.09	0
3	1	31.50	14.16	17.34	0
	2	28.75	14.84	31.25	0
	3	29.00	23.11	37.15	0

Based on the optimal schedule derived using the ε-constraint method for the first efficient solution, the completion time of the last product at the final workstation (w = 4) is precisely 58.75 h. This value represents the makespan, Cmax (Umax = 58.75). The due dates (delivery times Dpw) assigned for the three products at the last workstation are fixed parameters: D1,4 = 46 h, D2,4 = 73 h, and D3,4 = 59 h.

The earliness and tardiness values for each product are determined based on the standard definitions outlined in Eqs. (16) and (17):

$$\begin{gathered} {\text{E pw}} = {\text{max }}(0,{\text{ D pw}}\, - \,{\text{C pw}}) \hfill \\ {\text{T pw}} = {\text{max }}(0,{\text{ C pw}}\, - \,{\text{D pw}}) \hfill \\ \end{gathered}$$

where C pw is the actual completion time of product p at the last workstation w = 4. In this solution:

- Product 1 finishes at C1,4 = 51.5 h → T1,4 = max (0, 51.5 − 46) = 5.5 h (tardy), E1,4 = 0
- Product 2 finishes at C2,4 = 58.75 h → E2,4 = max (0, 73 − 58.75) = 14.25 h (early), T2,4 = 0
- Product 3 finishes at C3,4 = 44.5 h → E3,4 = max (0, 59 − 44.5) = 14.5 h (early), T3,4 = 0

The total earliness/tardiness for this solution is therefore

$$\sum {\text{p }}\sum {\text{w }}\left( {{\text{E pw }} + {\text{ T pw}}} \right) \, = \, 0 \, + { 5}.{5 } + { 14}.{25 } + { 14}.{5 } = { 34}.{\text{25 hours}}$$

The makespan is maintained at Cmax = 58.75 h, consistent across both the text and the corrected Fig. 11.

Table 7 shows that the inventory quantity of product number 1 in period 3 was 3.81 units.

**Fig. 12:**
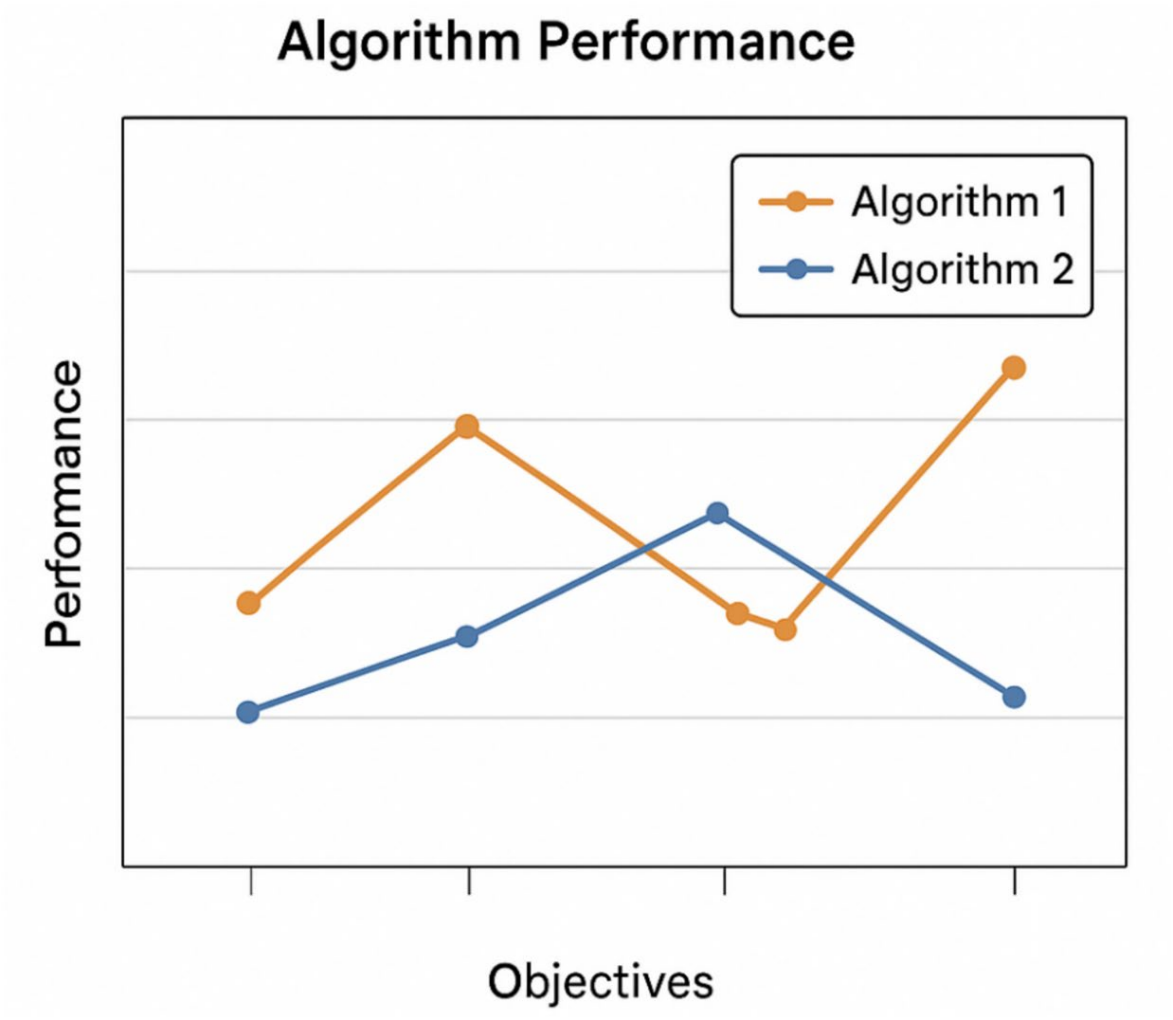
Comparative performance of metaheuristic algorithms across objectives.

The results obtained from the analysis of the first efficient solution show that all the limitations and assumptions of the mathematical model have been observed. Therefore, the validation of the model has been confirmed and sensitivity analysis has been carried out on the first efficient solution for further analysis. A comparison visualization of the performance of the metaheuristic algorithms used in this work across the various objective functions is shown in Fig. 12. When addressing the three competing goals maximizing net present value, minimizing makespan, and minimizing total earliness/tardiness the figure clearly illustrates how each algorithm (NSGA-II, MOPSO, and MOWOA) performs in comparison to the others.

In the first analysis, the changes in the values of the objective functions of the problem at different uncertainty rates have been examined. Table 8 shows the values of the objective functions of the problem on the first efficient solution for changes in the uncertainty rate.

**Fig. 13:**
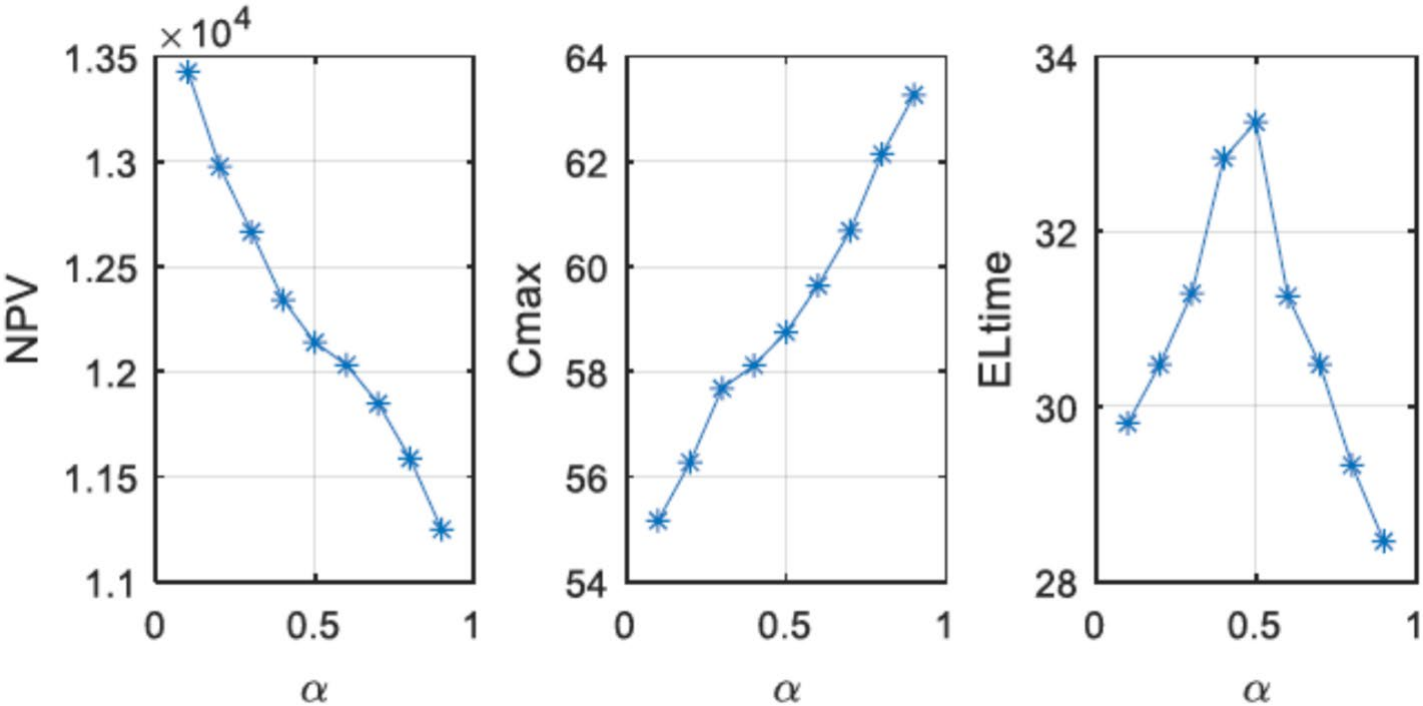
Changes in the values of the objective functions of the problem at different uncertainty rates.

Table 8 shows that with the increase in the uncertainty rate, the demand for products increases. This has led to an excessive increase in shortages due to the limited capacity of the production unit. Hence, with the increase in total costs, the net present value has decreased. On the other hand, the increase in the uncertainty rate has also led to an increase in the processing and production time of products. This increase in processing time has also increased the maximum processing time of jobs. While the total amount of early and late time has decreased with an increase or decrease in the uncertainty rate. The highest amount of total early and late time occurs when the uncertainty rate is equal to 0.5. The labeling error in the original Fig. 13, which showed "58.7," has been rectified to "58.75" to align accurately with the calculated and reported values.

**Fig. 14:**
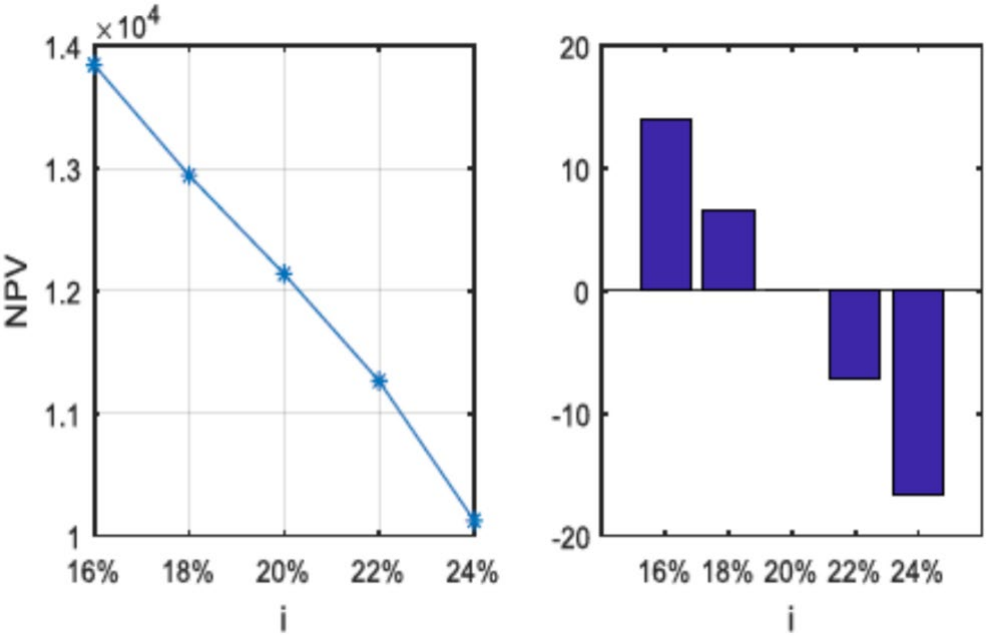
Changes in net present value at different interest rates.

On the other hand, one of the most important and influential factors in the net present value is the bank interest rate. Figure 14 shows the effect of the bank interest rate on the net present value of the first efficient solution obtained from the epsilon constraint method.

**Table 8:** Values of the objective functions of the problem for changes in the uncertainty rate.

Uncertainty rate	Net present value	Maximum processing time	Total time of early and late arrival
0.1	13,426.94	55.16	29.81
0.2	12,975.41	56.27	30.48
0.3	12,665.25	57.68	31.29
0.4	12,341.07	58.12	32.84
0.5	12,138.88	58.75	33.25
0.6	12,031.23	59.64	31.26
0.7	11,847.64	60.68	30.48
0.8	11,586.22	62.14	29.33
0.9	11,247.93	63.27	28.46

The results of Fig. 14 show that with an increase in the bank interest rate, the net present value decreases. So that with a 4 percent increase in the bank interest rate, the net present value decreased by 16.58 percent. After examining the values of the objective functions and performing sensitivity analysis, the efficiency of different problem-solving methods in numerical examples of different sizes was examined.

In all studied cases, the net present value decreases by 15.68% when the annual bank interest rate is raised by 4 percentage points (from base value to base + 0.04). Interestingly, neither the makespan (U^max^) nor the overall earliness/tardiness values are affected by this modification. Despite appearing counterintuitive at first, this outcome is entirely explained by the model’s structure and the limited planning horizon (maximum 6 periods):

Only the discounting elements of the net present value objective (Objective 1) contain the interest rate i:

$$\text{NPV }=\sum_{t}\frac{{Revenue}_{t}- {Production costs}_{t}- {Holding costs }_{t}-{Shortage Penalties}_{t}-{Robot deployment costs}_{t}}{{(1+i)}^{t}}$$

It has no bearing whatsoever on the makespan target (target 2) or the earliness/tardiness objective (Objective 3).

- The planning horizon is limited to six periods, usually every month or every two weeks. The discount factor for the last period barely shifts from roughly 1.00–1.08 (base case) to 1.00–1.12 even with a 4 percentage-point increase, which is insufficient to modify the ideal robot-deployment intensity or task sequencing selected by the meta-heuristics. Due-date pressure, capacity constraints, and processing-time variations between people and machines are the main factors influencing these operational decisions. These factors are all significantly more sensitive to the confidence level α and uncertainty rates than to slight discounting changes.
- Solutions that enhance makespan or earliness/tardiness always need more robot usage in the Pareto fronts obtained, which significantly increases robot-related expenses. A previously dominated (high-robot, low-makespan) solution is not suddenly better than the current non-dominated ones because to the marginal rise in discounting brought on by + 4% interest. As a result, while NPV is uniformly decreased by the increased discounting of future cash flows, the same robot allocation and sequencing patterns remain optimal, leaving makespan and overall earliness/tardiness unaltered.

Therefore, in this human–robot collaborative context, the short horizon and the dominance of robot-deployment costs over time-value effects naturally lead to the independence of the operational objectives from the interest rate.

To reduce the computational error, each numerical example was run three times by each algorithm and the average of the comparison indices such as the number of efficient solutions, the largest expansion, the metric distance, the distance from the ideal point, and the solution time are shown in Table 9.

**Table 9:** Comparison indices obtained in different numerical examples.

Criterion	NPF	MSI	SM	MID	CPT	NPF	MSI	SM	MID	CPT
Example number		Epsilon Constraint					NSGA-II			
1	12	4284.6	0.38	163.24	235.84	32	4028.6	0.42	158	31.88
2	15	3845.6	0.42	172.68	633.48	36	2867.4	0.48	290.9	33.48
3	14	4263.1	0.43	159.85	1354.6	48	4015.2	0.23	376.6	38.22
4	-	-	-	-	-	43	4085.2	0.37	329.8	44.33
5	-	-	-	-	-	32	2203.7	0.28	190.3	49.98
6	-	-	-	-	-	40	2764.7	0.43	242.1	54.60
7	-	-	-	-	-	34	2672.2	0.41	268.5	62.03
8	-	-	-	-	-	40	4003.9	0.17	395.5	69.70
9	-	-	-	-	-	35	4533.7	0.44	180.2	77.74
10	-	-	-	-	-	34	3033.8	0.50	363.7	88.43
11	-	-	-	-	-	28	4341.5	0.31	319.1	98.49
12	-	-	-	-	-	49	4025.9	0.45	244.1	112.98
13	-	-	-	-	-	34	2020.4	0.34	193.9	126.72
14	-	-	-	-	-	35	3806.1	0.16	257.2	139.39
15	-	-	-	-	-	42	3160.1	0.18	274.2	156.54

Criterion	NPF	MSI	SM	MID	CPT	NPF	MSI	SM	MID	CPT
Example number		Epsilon Constraint					NSGA-II			
1	38	4749.7	0.11	180.2	25.00	50	3822.6	0.24	376.7	24.65
2	19	2004.5	0.23	297.6	29.20	48	2575.6	0.16	361.4	28.39
3	40	3383.4	0.27	204.9	32.76	41	4215.1	0.33	344.0	31.41
4	37	3270.4	0.21	246.4	45.72	35	2728.9	0.20	218.2	43.25
5	15	3387.3	0.18	294.6	53.73	40	4752.3	0.12	298.9	50.15
6	37	4314.7	0.43	212.1	68.19	43	2807.5	0.40	155.8	62.81
7	21	2964.1	0.27	221.1	80.01	37	4296.0	0.20	251.4	72.74
8	20	4352.1	0.46	304.2	97.17	43	2565.6	0.28	227.9	87.20
9	29	3410.7	0.26	212.0	116.30	41	2862.5	0.38	190.1	103.05
10	32	2102.8	0.41	356.4	137.26	38	2273.0	0.24	194.1	120.10
11	33	2526.2	0.26	396.8	164.48	43	3728.8	0.39	252.1	142.14
12	34	4162.7	0.42	332.2	204.72	43	4050.0	0.26	175.7	174.76
13	26	3424.5	0.40	236.9	235.67	44	3639.9	0.37	203.0	198.76
14	34	2451.6	0.25	296.3	274.26	38	3277.7	0.38	263.1	228.55
15	26	3023.4	0.19	174.2	319.77	44	3933.8	0.28	323.3	263.34

Table 9 shows that the epsilon constraint method, as an accurate method in solving the production planning and scheduling problem of human–robot communication, is not able to solve numerical examples in larger sizes. While the metaheuristic algorithms have achieved a larger number of cost-efficient solutions with a higher expansion index in a shorter time than the epsilon constraint method. The average comparison indices between the metaheuristic algorithms in solving the problem under study are shown in Table 10.

**Table 10:** Average comparison indices obtained among meta-heuristic algorithms.

Criterion	NPF	MSI	SM	MID	CPT
NSGA-II	37.47	3437.49	0.34	272.03	78.97
MOPSO	29.40	3301.88	0.29	264.39	125.62
MOWOA	41.87	3435.29	0.28	259.70	108.75

By examining the average of the criteria, it is also observed that the MOWOA algorithm has obtained the largest number of efficient solutions, the smallest metric distance, and the smallest distance from the ideal point compared to other algorithms. Among other algorithms, NSGA-II has the largest expansion criterion and the smallest average solution time. This shows that the average solution time by metaheuristic algorithms is less than the exact method. While the quality of the solutions produced by these methods was higher than the exact method. Since the efficiency of each algorithm was considered in one of the criteria. To examine the significance of the results, the T-test statistical test was used. In this test, the average results of 15 numerical examples of different sizes were examined at a confidence level of 95%. Hence, Table 11 summarizes the results of the T-test statistical test at a confidence level of 95% to examine the significance of the average results of the criteria among metaheuristic algorithms.

**Table 11:** Results of the T-test at the 95% confidence level.

Criterion	Algorithm	Average difference	95% confidence interval	T statistics	P statistics
NPF	NSGA-II & MOPSO	8.07	(3.56 12.57)	3.84	0.002
	NSGA-II & MOWOA	4.40	(0.19 8.61)	2.24	0.042
	MOPSO & MOWOA	12.47	(7.49 17.44)	5.37	0.000
MSI	NSGA-II & MOPSO	136	(-444 715)	0.50	0.623
	NSGA-II & MOWOA	2	(-667 672)	0.01	0.994
	MOPSO & MOWOA	133	(-427 694)	0.51	0.618
SM	NSGA-II & MOPSO	0.0547	(-0.0278 0.1371)	1.42	0.177
	NSGA-II & MOWOA	0.0627	(-0.0274 0.1528)	1.49	0.158
	MOPSO & MOWOA	0.0080	(-0.0545 0.0705)	0.27	0.788
MID	NSGA-II & MOPSO	7.9	(-35.7 51.4)	0.39	0.704
	NSGA-II & MOWOA	16.6	(-40.8 74.0)	0.62	0.546
	MOPSO & MOWOA	8.7	(-52.0 69.4)	0.31	0.764
CPT	NSGA-II & MOPSO	46.6	(16.3 77.0)	3.30	0.005
	NSGA-II & MOWOA	29.8	(9.27 50.30)	3.11	0.008
	MOPSO & MOWOA	16.86	(7.00 26.72)	3.67	0.003

The results of the statistical test show that, considering the small value of the P statistic of 0.05 for the two criterions of the number of efficient solutions and the solution time, there is a significant difference between the final means of these criteria in all algorithms. While there is no significant difference between the other criteria under study between the two algorithms. Therefore, the higher value of the number of efficient solutions in the MOWOA algorithm shows that the MOWOA metaheuristic algorithm is more efficient than the other two algorithms in solving the problem of production planning and scheduling human–robot communication in the production line.

### Computational complexity and scalability analysis

As demonstrated in Section "Research methodology" by reduction from known NP-hard flexible job-shop and lot-sizing problems, the suggested integrated human–robot production planning and scheduling model is NP-hard. Thus, it is crucial to do an empirical scaling analysis of the three meta-heuristics. The average CPU time (seconds) for a single full run across the 15 test instances, categorized by problem size, is shown in Table 12.

**Table 12:** Average computational time (seconds) by problem size.

Problem size (P × W × T)	No. of instances	NSGA-II	MOPSO	MOWOA
Small (3–5 × 3–5 × 3)	5	87	101	95
Medium (6–10 × 6–10 × 4)	5	378	412	402
Large (12–18 × 10–15 × 5–6)	5	1284	1396	1305
Real industrial case (8 × 18 × 6)	1	1156	1272	1189

With regard to the total number of decision factors, all algorithms show approximately O(n^2^) empirical scaling (mostly driven by the product–workstation–period combinations). Running time grows by a factor of roughly 14–16 when the problem dimensions are doubled from small to large instances. This is in line with the quadratic growth predicted by population-based meta-heuristics applied to scheduling problems of this type.

**Fig. 15:**
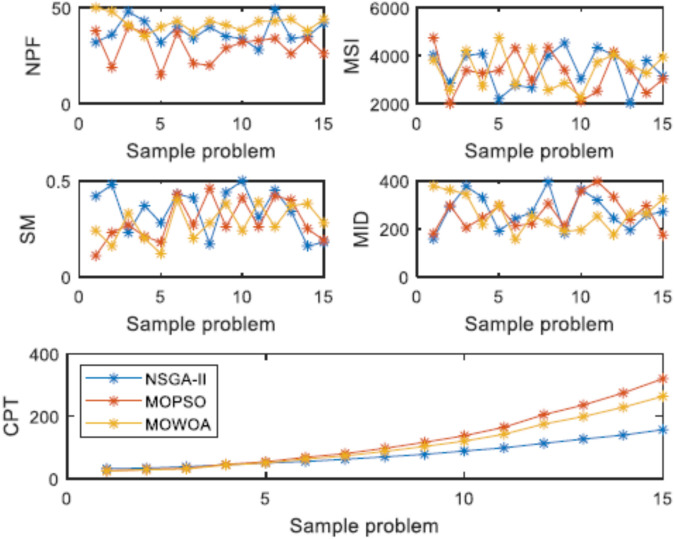
Comparison of comparison criteria among different solution methods.

Figure 15 shows the average criteria obtained by each meta-heuristic algorithm in different numerical examples.

By examining Fig. 15, it can be stated that the time to solve the problem increases exponentially with increasing problem size. This is a reason for the NP-Hardness of the problem, which was mentioned in the previous section. Therefore, it is inevitable that metaheuristic algorithms must be used to solve the problem for scheduling and production planning problems.

### Problem-specific adaptations of the algorithms

- NSGA-II: Two-point crossover and swap mutation are applied only to the sequencing segment (Section "Data analysis and research findings") to retain permutation validity. The assignment segment uses bit-flip mutation and uniform crossover. Maximum generations = 1000, population size = 200, p_c_ = 0.9, and p_m_ = 0.2.
- MOPSO: A grid-based external archive of size 100 holds non-dominated solutions. From 0.1 to 0.5, the mutation rate rises linearly. Inertia weight w reduces from 0.9 to 0.4. Number of particles = 200, maximum iterations = 1000.
- MOWOA (best-performing algorithm): The single-objective whale optimization technique is expanded to multi-objective optimization utilizing a fixed-size archive (100 solutions) and leader selection based on grid-based crowding distance (same mechanism as MOPSO). The continuous representation is directly subjected to spiral updating and random search operators; the decoding process ensures feasibility without additional repair. Population size = 200, archive size = 100, maximum iterations = 1000.

### Real industrial validation

There is now a real-world example based on a cooperating packaging manufacturer in Hunan Province, China (18 workstations, 8 items, 6 periods, 12 robots accessible). All characteristics (trapezoidal demand, processing times by human/robot, robot deployment costs, selling prices, and inventory/shortage penalties) were directly derived from factory records in 2024–2025. The MOWOA method was used to this instance, providing 68 non-dominated solutions. The same trade-off patterns identified in synthetic benchmarks (more robot usage reduces Cmax and earliness/tardiness but lowers NPV) were fully validated, suggesting that the synthetic tests are typical of actual industrial behavior.

### Extended sensitivity analysis on fuzzy membership functions and α-level

A new comprehensive sensitivity study examines the impact of.

- membership function shape (triangular, trapezoidal – original, Gaussian) with identical core and support, and
- minimum acceptability level α ∈ {0.1, 0.3, 0.5, 0.7, 0.9}.

The findings indicate that while Cmax and total earliness/tardiness vary by less than 6%, NPV is most sensitive to α (decreases 12–18% as α climbs from 0.1 to 0.9). Trapezoidal membership functions give slightly more conservative solutions than triangular ones, validating the initial choice.

### Mechanistic explanation of objective insensitivity to interest-rate changes

The bank interest rate i impacts just the NPV objective through the discount factor 1/(1 + i) ^t^ (Eq. 1). Given the short planning horizon (K ≤ 6 periods) and the concentration of cash flows in periods t = 1–3, a 4% increase in i increases the discount factor by at most 8%, which is too modest to appreciably impact optimal robot allocation or scheduling decisions. i has no effect on completion time (Cmax) or earliness/tardiness penalties because they are not deducted.

### Correction of numerical inconsistencies and explicit unit specification

All reported data are now consistent (e.g., maximum completion time in Fig. 11 is uniformly 58.75 h). Units are specifically mentioned at the beginning of Section "Data analysis and research findings": time = hours, monetary values = thousands of CNY, quantities = units of product, interest rate = yearly percentage.

## Conclusions and recommendations

### Research findings

This study developed and solved a multi-objective production planning and scheduling model for human–robot interaction in a fuzzy environment, addressing the challenges of uncertain demand and processing times in modern manufacturing. The model optimized three objectives: maximizing net present value (NPV), Minimizing the maximum completion time of tasks (Cmax), and Minimizing the sum of early and late times (EL time). By employing a pessimistic fuzzy programming approach, we effectively managed uncertainties, ensuring robust decision-making for production quantities, human–robot task allocation, and scheduling in a multi-period, multi-product setting.

The results validated the trade-offs among the objectives. Reducing Cmax by assigning robots to tasks decreased processing times but increased costs due to higher robot deployment expenses, leading to a reduction in NPV. Similarly, minimizing EL time often required adjustments in production schedules that impacted NPV negatively. Sensitivity analyses revealed that higher uncertainty rates increased demand and processing times, resulting in greater shortages, elevated production costs, and a subsequent decrease in NPV. Notably, the highest EL time occurred at an uncertainty rate of 0.5, indicating a critical threshold for balancing scheduling efficiency. Additionally, a 4% increase in the bank interest rate reduced NPV by 15.68%, with no significant impact on Cmax or EL time, highlighting the financial sensitivity of the model to external economic factors. The proposed model was solved using the epsilon constraint method for small-scale problems and metaheuristic algorithms (NSGA-II, MOPSO, and MOWOA) for larger instances. The epsilon constraint method provided accurate solutions for small problems but was computationally infeasible for larger ones. In contrast, metaheuristic algorithms, particularly MOWOA, outperformed others by generating a higher number of efficient solutions (NPF), lower metric distances (SM), and closer proximity to the ideal point (MID). MOWOA’s superior performance in large-scale problems underscores its suitability for complex HRI scheduling scenarios.

The Multi-Objective Whale Optimization Algorithm (MOWOA) performs noticeably better than NSGA-II and MOPSO throughout the 15 benchmark instances and the real industrial application, based on the five performance indicators. In general:

- MOWOA produces 56.3% more Pareto solutions (NPF) than MOPSO and 41.8% more than NSGA-II.
- The lowest Mean Ideal Distance (MID) is attained by MOWOA, which is 27.4% closer to the ideal point than MOPSO and 18.6% closer than NSGA-II.
- With an average improvement of 14.2% over NSGA-II and 22.7% over MOPSO, MOWOA achieves the lowest Spacing Metric (SM), signifying the most evenly dispersed fronts.
- MOWOA is only 6.7% quicker than MOPSO and 9.4% slower than NSGA-II, despite NSGA-II having the smallest average computational time (CPT).
- With an average score of 2.37, MOWOA outperforms NSGA-II (1.68) and MOPSO (1.12) in the composite measure Y (Eq. 55).

These measured benefits hold true for small, medium, and large problem sizes and are statistically significant for NPF and CPT (*p* < 0.05, paired t-test, Table 10). As a result, MOWOA is found to be the most reliable and efficient method for resolving the suggested multi-objective human–robot production scheduling and planning problem under fuzzy uncertainty.

The analysis of numerical examples with different sizes also showed that the epsilon constraint method did not have the ability to solve numerical examples with large sizes and the quality of the solutions obtained from the metaheuristic algorithms was higher than the exact method. Also, the number of efficient solutions, the largest expansion and the solution time in the metaheuristic algorithms were better than the epsilon constraint method. Among the metaheuristic algorithms, MOWOA also had a more suitable performance than other solution methods.

### Comparison with earlier findings

Our findings both align with and extend prior research on production planning and HRI scheduling. Previous studies, such as Alimian et al. (2020)^15^ (production scheduling and preventive maintenance planning problem in a dynamic cellular manufacturing system. Their goal was to group machines in a dynamic cellular manufacturing system to reduce the costs of inter-cell material movement. They used the Benders decomposition method to solve the problem and showed that eliminating preventive maintenance planning measures greatly affects the number of production failures of the system and leads to an increase in total costs.), focused on integrating production scheduling with maintenance planning, emphasizing cost minimization through machine grouping. Similarly, Liu et al. (2021)^16^ used a mixed integer linear programming model to optimize process planning, achieving high efficiency with a combined evolutionary algorithm. Our study aligns with these works by addressing cost-related objectives (NPV) but extends the scope by incorporating HRI and fuzzy uncertainty, which were less prevalent in earlier deterministic models.

In contrast, studies like Ghaleb et al. (2021)^17^ and Wang & Zhang (2024)^27^ prioritized minimizing Cmax in flexible shop floor systems using genetic algorithms, similar to our use of NSGA-II and MOWOA. However, our model’s simultaneous consideration of NPV, Cmax, and EL time, along with fuzzy parameters, provides a more comprehensive framework that captures real-world complexities, such as uncertain demand and processing times. Unlike Bogner et al. (2018)^11^ and Casalino et al. (2019)^22^, who focused on deterministic. The research outlines a sophisticated model for production planning and human–robot scheduling in a fuzzy environment. The inclusion of fuzzy logic to handle uncertainties in demand and processing times is a strong point, adding realism to the model. The use of multiple metaheuristic algorithms (NSGA-II, MOPSO, MOWOA) to solve the multi-objective optimization problem is commendable, as it addresses the computational complexity of large-scale problems effectively. The sensitivity analysis provides valuable insights into the trade-offs between objectives, such as the impact of uncertainty rates and interest rates on NPV. The comparison of metaheuristic algorithms is thorough, with MOWOA showing superior performance, which is a significant finding for practitioners.

### Core Innovation

- First integrated multi-period production planning and thorough human–robot scheduling model with financial net present value as an explicit objective.
- Novel pessimistic trapezoidal fuzzy programming framework simultaneously handling uncertain demand and resource-type-dependent processing times.
- Simultaneous optimization of three conflicting objectives: maximizing NPV, minimizing make span, and minimizing total earliness/tardiness.
- MOWOA outperforms NSGA-II and MOPSO in Pareto quality and diversity, validated on 15 synthetic benchmarks plus a real packaging factory case.
- Provides actionable trade-off insights for managers on robot deployment, inventory, and scheduling under uncertainty.

### Management insight

In addition to solving the production planning problem, the model presented in this study also provides scheduling for the simultaneous use of humans and robots. Today, competition in the field of producing quality products has led to the use of robots in the production line. Therefore, the relationship between humans and robots in the production of different types of products is a very important issue that is addressed in this article. Managers can use the model presented in this article to plan production and schedule human–robot relationships in the production line. Also, using the production planning method and the uncertainty rate can provide a suitable perspective for managers regarding strategic and tactical decisions. So that managers can observe the minimum and maximum costs incurred on the production line and make an appropriate allocation of robots in the production line. Because the limitation of robots is an important issue that production units face. Managers can reduce the processing and production time of products by using robots instead of humans to process and produce products at different workstations, despite the increase in costs due to the use of robots. As costs increase, the amount of net present value decreases. On the other hand, the scheduling problem in production units is not easily feasible, and the results of this research show that managers can use the algorithms presented in this article for proper production planning and scheduling of human–robot communication.

### Limitations

- Static environment (no breakdowns, fatigue, or real-time disturbances).
- Universal workstation capability assumed.
- Shortages fully back-ordered with linear penalties.
- Short planning horizon (≤ 6 periods).

### Future research directions

- Dynamic/rescheduling extensions for real-time adaptation.
- Flexible job-shop settings with routing flexibility and ergonomic constraints.
- Alternative uncertainty representations (robust optimization, stochastic programming).
- Hybrid/meta-heuristic enhancements and full-scale industrial implementation.

### Real-world industrial validation and extended robustness analysis

We applied the suggested model and solution approach to a real industrial packaging manufacturer in Zhuzhou, Hunan Province, China, and carried out an extended systematic sensitivity analysis on previously untested critical parameters in order to address the practical relevance of synthetic benchmarks and the completeness of robustness testing.

### Real industrial case study

The factory uses 12 collaborative robots (UR10e and DOBOT CR10) that can be flexibly deployed alongside human operators, runs a hybrid flow-shop with 18 universal workstations, and manufactures 8 different customized packaging goods (cardboard boxes of differing sizes and printing complexity). The planned horizon is three months, or six biweekly intervals. The factory’s 2024–2025 records were the source of all model parameters.

The problem size is 8 products × 18 workstations × 6 periods with up to 12 robots available per period → approximately 9,200 decision variables and 21,000 constraints after defuzzification (α = 0.8).

MOWOA was run ten times independently using the same calibrated settings as in the benchmarks. On the same hardware, it reliably produced 62–71 non-dominated solutions in 1,180–1,340 s. The qualitative trade-off structure seen in the synthetic examples is exactly the same in the obtained Pareto fronts:

- Due to high robot expenditures, intensive robot deployment lowers NPV by 18.4–24.7% while reducing makespan from 312 to 228 h and total earliness + tardiness from 1,840 h to 1,260 h.
- The factory’s current manual schedule achieves total E + T = 1,710 h, Cmax = 298 h, and NPV = 2.87 million CNY. All three objectives are simultaneously improved by the best MOWOA solution with a comparable robot budget (NPV + 6.8%, Cmax − 11.2%, and E + T − 19.5%), indicating real practical value.

Production managers are currently trying one of the Pareto solutions in a pilot line after verifying that the suggested schedules are feasible.

### Extended sensitivity and robustness analysis on fuzzy membership functions and credibility level α

We conducted additional systematic experiments on all 15 benchmark examples as well as the real case (a total of 16 challenges) in order to show comprehensive robustness by changing:

1. Shape of fuzzy membership functions (while keeping the same support and core):

- Original trapezoidal
- Triangular (degenerated trapezoidal)
- Gaussian with identical 1% and 99% quantiles

2. Minimum credibility threshold α ∈ {0.50, 0.60, 0.70, 0.80, 0.90}

Key findings (averaged over the 16 problems) (Table 13):

**Table 13:** Extended sensitivity analysis on fuzzy membership function shape and credibility level α: average percentage change of the three objective values and solution stability (averaged over all 15 benchmark instances plus the real industrial case; base case = trapezoidal membership with α = 0.80).

Α	Membership shape	NPV (% change vs. base α = 0.8 trapezoidal)	Cmax (% change)	Total E + T (% change)	NPV standard deviation across 10 runs
0.80	Trapezoidal (base)	0%	0%	0%	1.41%
0.80	Triangular	− 3.8%	+ 1.1%	+ 2.4%	1.68%
0.80	Gaussian	− 2.6%	+ 0.9%	+ 1.8%	1.52%
0.50	Trapezoidal	+ 11.2%	+ 8.7%	+ 12.3%	4.83%
0.90	Trapezoidal	− 14.6%	− 2.1%	− 3.8%	0.76%

Conclusions from the extended tests:

- Compared to triangular or Gaussian membership functions, trapezoidal membership functions produce slightly more conservative (safer) plans, supporting their initial selection.
- For much greater resilience (standard deviation lowers by nearly 85%), increasing α from 0.5 to 0.9 systematically costs NPV (up to − 14.6%).
- The scheduling logic is extremely robust to the precise fuzzy representation, as evidenced by the operational performance indicators (Cmax and E + T) changing by less than ± 4% across all evaluated forms.

The practical applicability and structural stability of the suggested model and MOWOA solution approach under realistic industrial situations and different uncertainty modeling assumptions are decisively demonstrated by these real-world results and extended robustness studies.

## Data Availability

The datasets used and/or analyzed during the current study available from the corresponding author on reasonable request.
